# Sandfly Fever Sicilian Virus-*Leishmania major* co-infection modulates innate inflammatory response favoring myeloid cell infections and skin hyperinflammation

**DOI:** 10.1371/journal.pntd.0009638

**Published:** 2021-07-26

**Authors:** Ellen Heirwegh, Emily MacLean, Jinlei He, Shaden Kamhawi, Selena M. Sagan, Martin Olivier

**Affiliations:** 1 Infectious Diseases and Immunology in Global Health, McGill University Health Centre, McGill University, Montréal, Québec, Canada; 2 China School of Basic Medical Sciences and Forensic Medicine, Sichuan University, Chengdu, China; 3 Vector Molecular Biology Section, National Institute of Health, Rockville, Maryland, United States of America; 4 Department of Microbiology and Immunology, McGill University, Montréal, Québec, Canada; Vienna, AUSTRIA

## Abstract

**Background:**

The leishmaniases are a group of sandfly-transmitted diseases caused by species of the protozoan parasite, *Leishmania*. With an annual incidence of 1 million cases, 1 billion people living in *Leishmania*-endemic regions, and nearly 30,000 deaths each year, leishmaniasis is a major global public health concern. While phlebotomine sandflies are well-known as vectors of *Leishmania*, they are also the vectors of various phleboviruses, including Sandfly Fever Sicilian Virus (SFSV).

Cutaneous leishmaniasis (CL), caused by *Leishmania major* (*L*. *major*), among other species, results in development of skin lesions on the infected host. Importantly, there exists much variation in the clinical manifestation between individuals. We propose that phleboviruses, vectored by and found in the same sandfly guts as *Leishmania*, may be a factor in determining CL severity. It was reported by our group that *Leishmania* exosomes are released into the gut of the sandfly vector and co-inoculated during blood meals, where they exacerbate CL skin lesions. We hypothesized that, when taking a blood meal, the sandfly vector infects the host with *Leishmania* parasites and exosomes as well as phleboviruses, and that this viral co-infection results in a modulation of leishmaniasis.

**Methodology/Principal findings:**

*In vitro*, we observed modulation by SFSV in MAP kinase signaling as well as in the IRF3 pathway that resulted in a pro-inflammatory phenotype. Additionally, we found that SFSV and *L*. *major* co-infection resulted in an exacerbation of leishmaniasis *in vivo*, and by using endosomal (Toll-like receptor) TLR3, and MAVS knock-out mice, deduced that SFSV’s hyperinflammatory effect was TLR3- and MAVS-dependent. Critically, we observed that *L*. *major* and SFSV co-infected C57BL/6 mice demonstrated significantly higher parasite burden than mice solely infected with *L*. *major*. Furthermore, viral presence increased leukocyte influx *in vivo*. This influx was accompanied by elevated total extracellular vesicle numbers. Interestingly, *L*. *major* displayed higher infectiveness with coincident phleboviral infection compared to *L*. *major* infection alone.

**Conclusion/Significance:**

Overall our work represents novel findings that contribute towards understanding the causal mechanisms governing cutaneous leishmaniasis pathology. Better comprehension of the potential role of viral co-infection could lead to treatment regimens with enhanced effectiveness.

## Author summary

The leishmaniases are a group of chronic infectious diseases caused by parasites of the genus *Leishmania*. The disease poses a great public health risk; millions of people are affected annually. In addition, leishmaniasis is characterized by widely varying clinical manifestations, for which the cause is still unknown. Parasites of the genus *Leishmania* are the ethological agents of the disease and are vectored by phlebotomine sandflies.

While examining sections of the sandfly midgut, we noted viral entities, later identified as phleboviruses. Interestingly, there is a large overlap in the geographic distribution of *L*. *major* and Sandfly Fever Sicilian Virus (SFSV); this led us to hypothesize that viral presence could influence disease severity. With the use of various wild-type and knock-out mouse models, we deduced that co-infection with SFSV indeed affected disease severity, leading to exacerbated skin lesions and higher parasite numbers. However, this was not paralleled by the modulation of immunomodulatory molecules. Our findings demonstrate that viral presence may facilitate parasite entry into host cells.

Overall, our research constitutes novel findings that contribute toward a better understanding of the mechanisms underlying leishmaniasis infections.

## Introduction

The leishmaniases are a group of sandfly-transmitted diseases caused by various species of the protozoan parasite *Leishmania*. With an estimated annual incidence of 0.7 to 1.2 million cases in 98 tropical and subtropical countries [1, 2], 1 billion people living at risk in *Leishmania*-endemic regions, and nearly 30,000 deaths each year due to visceral leishmaniasis (VL), this parasitic infection is a major global public health concern [2]. Cutaneous leishmaniasis (CL), caused by *L*. *major* and other species, results in development of skin lesions on the infected host. Importantly, there exists much variation in CL severity and progression between individuals, with many possible complications in pathology [3].

From North Africa to the Middle East, *L*. *major* is transmitted by the bite of the phlebotomine sandfly, predominantly the species *Phlebotomus papatasi* (*P*. *papatasi*) [4]. When taking a blood meal, the sandfly regurgitates *L*. *major* and other material, including *Leishmania* exosomes, into the host [5, 6]. We have previously established that co-egested exosomes exacerbate CL skin lesions by overproducing inflammatory cytokines known to drive the Th17 immune response involved in various skin pathologies (e.g. psoriasis) [7]. Additionally, it has now been documented that other infectious material may be transmitted along with *Leishmania* [8].

*P*. *papatasi* also serves as a vector for a less familiar group of pathogens, the phleboviruses, which, like *L*. *major*, are found in the sandfly gut [9]. These viruses, classified as the genus *Phlebovirus*, family *Phenuiviridae* [10], are 80–120 nm in diameter [11] and contain a tripartite single-stranded RNA genome, composed of the S, M, and L segments [12]. Rift Valley Fever Virus, the most well-known of the genus, causes a highly pathogenic febrile illness that in 1–2% of humans becomes a fatal hemorrhagic fever [11]. With very few exceptions, other phleboviral diseases are not as lethal, with most viruses producing an approximately 3-day illness comprised of fever, headache, and vomiting [13].

The geographic distribution of sandfly-borne phleboviruses was reviewed by Alkan and colleagues; these viruses are found from southern Europe through Turkey, to the Middle East and North Africa, into Pakistan [1, 9]. Interestingly, *L*. *major* and Sandfly Fever Sicilian Virus (SFSV) have almost completely overlapping geographic distributions, and in *Leishmania*-endemic areas, SFSV seroprevalence of sampled populations ranges from positive rates of 1% in France to 36% in Turkey and 56% in Egypt [9].

As they are commonly vectored, this raises the possibility that parasites of the genus *Leishmania* and sandfly-borne phleboviruses are co-injected into a host during the sandfly bloodmeal. Since it has been well-established that other viral presence may influence disease severity and clearance [14], the presence of a coincident phleboviral infection could, ultimately, be a contributing factor that explains the wide range of observed CL pathologies in the Old World.

In this study, we document the role of SFSV in exacerbated *L*. *major*-induced CL *in vivo*, and with several wild-type and knock-out mouse models, propose the mechanisms through which this enhancement occurs. Ultimately, we determined that SFSV and *L*. *major* co-infection exacerbates CL because of a host immunopathological response that occurs through TLR3- and MAVS-dependent pathways. Additionally, as we observed increased initial cell recruitment and infective parasite load, we established that initial inflammatory responses and early interactions between parasites and immune myeloid cells appear crucial. This was reflected in elevated numbers of extracellular vesicles, which have potential pro-inflammatory properties.

## Methods

### Ethics statement

Animal experiments were performed in compliance with the Canadian Council on Animal Care (CCAC) Guidelines and policies, as approved by the Institutional Animal Care and Use Committees at McGill University, under ethics protocol numbers 4859 and 7791.

### Cells and antibodies

Immortalized bone marrow-derived macrophages from B10A.Bcg^r^ mice (B10R macrophages) were grown and maintained at 37°C in 5% CO_2_ in Dulbecco’s Modified Eagle’s Medium (Wisent Inc., St-Bruno, QC) supplemented with 10% heat-inactivated fetal bovine serum (FBS) (Invitrogen, Burlington, ON, Canada), 2mM L-glutamine, 100U/ml penicillin, and 100 μl/ml streptomycin (Wisent, St-Bruno, QC, Canada). BHK-21 cells (ATCC) were grown and maintained at 37°C with 5% CO_2_ in Eagle’s Minimum Essential Medium (Wisent) supplemented with 5% heat-inactivated FBS, 2mM L-glutamine, 100 U/ml penicillin, and 100 μl/ml streptomycin.

Primary antibodies purchased for immunoblotting were phospho-p44/42 (ERK 1/2) (#9101S); ERK 1/2 (#4695S); phospho-p38 (#9216S); p38 (#9212S); phospho-IRF3 (4D4G); IRF3 (D6I4C), phospho-TBK1/NAK (D52C2), and TBK (D1B4) (Cell Signaling Technology, Danvers, MA, USA).

### Parasite culture

*L*. *major* strain NIH S (MHOM/SN/74/Seidman) clone A2, generously supplied by Dr Shaden Kamhawi of the NIH, was cultured at 25°C in Schneider’s Drosophila Medium (Gibco-BRL, Grand Island, NY) supplemented with 10% heat-inactivated FBS, 5mg/ml HEMIN, 2 mM L-glutamine, 100 U/m penicillin, and 100 μl/ml streptomycin and passaged biweekly. Cultures of promastigotes were passaged at least twice and then grown to stationary phase for infection in all *in vitro* and mouse experiments.

### Virus culture

As indicated by the manufacturer, BHK-21s were infected with SFSV (ATCC, Manassas, VA, USA) when 30–50% confluency was reached, at an MOI of 0.1. After infection, cells were left for 4 days and passaged 3 or 4 times before collection of supernatant 5 days after the final passage. Supernatants were filtered through 0.2 μm filters (Millipore), titrated by quantitative reverse transcriptase PCR, and aliquots were stored at -80°C.

### Virus titration by Quantitative reverse transcription polymerase chain reaction (qRT-PCR)

As we encounterd some challenges utilizing the plaque assay, we decided to titrate the virus by qRT-PCR. Therefore, the pGEM-T vector, expressing plasmid DNA coding for the NS-s gene for the non-structural protein, was used to obtain a standard curve. A dilution series of the plasmid was prepared from a known number of copies and primers were designed to amplify the NS-s gene, segment S (Phleb-qPCR-F1: 5’ tcattgacccaagcctcgac 3’, Phleb-qPCR-R1: 5’ gccctcctctgagtgttgtc 3’). After amplification by qRT-PCR, a standard curve was designed (S1 Fig).

Total RNA from the supernatant of infected BHK-21s was extracted with TRIzol reagent (Life Technologies, Rockville, MD, USA), and DNA contaminants removed by RQ1 DNAse (Promega, Madison, WI, USA) treatment; samples were purified using the RNeasy Mini Kit (Qiagen, Venlo, Netherlands). Three to 5 μg of RNA were reverse transcribed by Protoscript II (New England Biolabs, Whitby, ON, Canada) and random primers (Carlsbad, CA, USA), RNAse H (New England Biolabs) was applied, and all templates were dosed and aliquoted to a standard concentration for use in qRT-PCR. Primer-BLAST was used to design primers listed above that were added to template and SYBR Green Supermix (Bio-rad), and qRT-PCRs were run in a CFX96 Touch Real-Time PCR Detection System (Bio-Rad).

### Sandfly infections

For parasitic infection, *Lutzomyia* sandflies were infected, and their gut lavages collected for TEM and proteomic analysis from the gut content as previously described [7].

### Transmission electron microscopy (TEM)

Gut lavages and vesicles were coated directly on formvar carbon grids (Agar Scientific Ltd, Stansted, Essex, UK). Gut lavages were fixed with 1% glutaraldehyde in 0.1 M sodium cacodylate buffer for 1 minute and stained with 1% uranyl acetate for 1 minute. Isolated vesicles were fixed with 2% glutaraldehyde (Millopore Sigma, Burlington, MA, USA) washed three times with autoclaved Milli-Q water and stained with 2% uranyl acetate for 1 minute each. For ultrastructure analysis, dissected midguts from infected or uninfected sandflies were fixed with 2.5% glutaraldehyde in 0.1 M sodium cacodylate buffer overnight. After washes in the same buffer, samples were post-fixed with osmium tetroxide and dehydrated in a graded acetone series prior to embedding in epoxy resin. Ultrathin sections (70–80 nm) were cut from resin blocks using a Reichert-Jung Ultracut E Ultramicrotome (Biel, Switzerland). Formvar grids covered with gut lavages or with ultrathin sections, as described above, were visualized in the FEI Tecnai 12 120 kV transmission electron microscope. Images were taken with the AMT XR-80C CCD Camera System. Intraperitoneal vesicle samples were visualized with the FEI Tecnai *G^2^* Spirit Twin 120kV Cryo-TEM and images were taken with the Gatan Ultrascan 4000 4kx4k CCD Camera System, model 895 (Facility for Electron Microscopy Research, McGill University, QC, Canada).

### Liquid chromatography-mass spectrometry (LC-MS/MS)

Liquid chromatography-tandem mass spectrometry (LC-MS/MS) on gut lavages was performed at the Institut de Recherches Cliniques de Montreal (IRCM, Université de Montréal), as described previously [7]. Proteins were precipitated with 15% trichloroacetic acid (TCA)/ acetone and digested with trypsin at a final concentration of 2 ng/ml. After an 18-hr incubation at 37°C and prior to the LC-MS/MS analysis, reactions were quenched by the addition of formic acid to a final concentration of 1%. The LC column was a PicoFrit fused silica capillary column (New Objective) self-packed with C-18 reverse-phase material (Phenomenex). The column was installed on the Easy-nLC II system (Proxeon Biosystems) and coupled to the Q Exactive mass spectrometer (Thermo Fisher Scientific), equipped with a Proxeon nanoelectrospray Flex ion source. The buffers used for chromatography were 0.2% formic acid (buffer A) and 100% acetonitrile/0.2% formic acid (buffer B). Peptides were loaded on the column at a flow rate of 600 nl/min and eluted with a two-slope gradient at a flow rate of 250 nl/min. Solvent B first increased from 2% to 40% in 85 min, and then from 40% to 80% in 25 min. LC-MS/MS data was acquired using a data-dependent top 15 method and standard values were used for all the parameters of the mass spectrometer.

### Protein database search

As previously described [7], individual sample tandem mass spectrometry spectra were peak listed using the Distiller version 2.1.0.0 software (www.matrixscience.com/distiller) with peak picking parameters set at 1 for signal noise ratio and at 0.3 for correlation threshold. The peak-listed data was then searched against the NCBI database with Mascot software, version 2.3.02 (Matrix Science, London, UK). Mascot was programmed to search the phleboviruses database (NCBI:txid11584 proteins) with a fragment ion mass tolerance of 0.50 Da and a parent ion tolerance of 1.5 Da. Carbamidomethyl was specified in both search engines as a fixed modification. Oxidation of methionine residues was specified in Mascot as a variable modification. Scaffold software version 4.0.6.1 (Proteome Software Inc., Portland, OR) was used to validate MS/MS peptide and protein identifications. Identification of peptides was accepted if it could be established at greater than 95.0% probability and contained at least 2 identified peptides. Proteins that contained similar peptides and could not be differentiated based on MS/MS analysis alone were grouped to satisfy the principles of parsimony.

### Mice

All mouse experiments were carried out in pathogen-free housing at McGill University according to CACC Guidelines and were approved by the McGill University Animal Care Committee. Male and female wild-type C57BL/6 mice were used from our in-house colony, or supplied by Charles River (Senneville, QC, Canada). Very generously, TLR3^-/-^ mice were provided by Dr. Silvia Vidal; Type 1 interferon receptors (IFNAR)^-/-^ mice were provided by Dr. Jörg Fritz; and MAVS^-/-^ mice were provided by Dr. Rongtuan Lin.

### Mouse infections

Mouse hind right footpads were infected with either 5 x 10^6^ stationary phase promastigotes, 2 x 10^4^ Sandfly Fever Sicilian virus particles (titrated by qRT-PCR), or 5 x 10^6^ stationary phase promastigotes and 2 x 10^4^ SFSV particles. Progression of cutaneous leishmaniasis was observed weekly, with measurements of the infected footpads taken up to 15 weeks post-infection; lesion size was defined as the difference in size between the infected and non-infected footpads.

For the study of innate inflammatory response, male C57BL/6 mice (6 to 7 weeks old), supplied by Charles River (Senneville, QC, Canada), were injected intraperitoneally with either endotoxin-free PBS (Wisent Inc, St-Bruno, QC) as a negative control, 4 x 10^5^ virus particles, 10^8^ stationary phase *L*. *major* promastigotes, or co-infected with 4 x 10^5^ SFSV particles and 10^8^ stationary phase *L*. *major* promastigotes. All injections were prepared using endotoxin-free PBS (Wisent Inc, St-Bruno, QC) with a final volume of 250 μl per injection. Per experiment, several mice were infected, the experiments were repeated at 3 independent occasions. Mice were sacrificed 6 hours post-infection.

### Footpad parasite load

In a manner previously described by our lab [7], *L*. *major-*infected C57BL/6 mice were sacrificed and footpads collected and sterilized with a chlorine dioxide disinfectant, before subsequent washing in 70% ethanol for 5 minutes. Footpads were further washed with PBS, dissected, and placed in a glass tissue homogenizer with 6 ml of PBS before manual homogenization until tissue disruption. Tissue homogenates were centrifuged at 3000 × *g* for 5 minutes, resuspended in PBS and titrated by Bradford assay. A limiting dilution assay was used to ascertain footpad parasite load. Volumes of the titrated homogenate were adjusted and 100 μl were added, in duplicate, to 96-well plates with wells containing 100 μl of Schneider’s Completed Drosophila Medium; for each duplicate, 24 2-fold dilutions were prepared. Plates were kept at 26°C and examined 6–10 days post-dilution; the highest dilution at which promastigotes were observed was noted, and parasite load was expressed as number of parasites per footpad.

### Peritoneal cavity lavage

Six hours post-intraperitoneal infection, mice were sacrificed and generously sprayed with 75% ethanol. The peritoneum was exposed and 5 ml of cold, endotoxin-free PBS (Wisent Inc, St-Bruno, QC, Canada) was injected in the peritoneal cavity, carefully avoiding organ perforation. After injection, the peritoneum was gently massaged to loosen attached cells into the PBS solution. Lavages were subsequently collected by carefully moving the needle in the peritoneal cavity while aspirating. Samples were kept on ice.

### Western blot

B10R macrophages were infected for 0.5 h, 1 h, and 3 h, with similar numbers of viral particles as in footpad injection, or stimulated with LPS (100 ng/ml) for 60 minutes as performed for induction of NF-κB nuclear translocation; uninfected wells served as negative controls. Experiments were performed in triplicate and proteins were extracted as previously described [15]. Briefly, following cell lysis and protein extraction of infected and uninfected cells, protein contents were dosed by Bradford Assay (Bio-Rad, Mississauga, ON, Canada). Proteins were resuspended in an SDS sample buffer containing bromophenol blue and beta-mercapto-ethanol, separated by SDS-PAGE using 10% acrylamide gels, and transferred to PVDF membranes (Perkin Elmer, Waltham, MA). Membranes were blocked for 1 hour in TBS- 0.05% Tween 20 containing 5% BSA before overnight incubation with the primary antibody (e.g. phospho-p44/42 (ERK 1/2), ERK 1/2, phospho-p38, p38, phospho-IRF3, IRF3, phospho-TBK1/NAK, TBK). Membranes were washed and incubated with the rabbit secondary anti-HRP-conjugated antibody (GE Healthcare, Baie d’Urfe, QC, Canada), and proteins were visualized by ECL Western Blot Detection System (GE Healthcare). USA).

### Cell count

Live cells collected through intraperitoneal lavages were immediately counted. Differential and infective cell counts were performed microscopically on a cytospin slide. Collected lavages were added onto the cytoslides, and cytospin preparations were centrifuged at 100 x g for 5 minutes. Subsequently, slides were stained using Wright-Giemsa staining (RAL diagnostics Diff-Quick kit) and air-dried.

### Multiplex cytokine and chemokine assay

Cytokine and chemokine levels in peritoneal lavage samples were determined using multiplex LASER bead assays. The 44-Plex cytokine and chemokine array was performed by EVE Technologies (Calgary, AB, Canada).

### Extracellular vesicle isolation

Peritoneal cavity lavages were centrifuged for 5 minutes at 620 x g and supernatant was collected. To increase the yield, supernatant of three mice was pooled together for each group before filtration through a 0.45 μm syringe filter (Millipore Sigma, Burlington, MA, USA). Next, exosomes were pelleted as previously described [16] by ultracentrifugation at 100,000 x g for 1 hour, and resuspended in exosome buffer (137 mM NaCl, 20 mM Hepes, pH 7,5). A second round of a 1-hour ultracentrifugation at 100,000 x g was carried out for further purification, and the pellet was resuspended in ~300 μl exosome buffer. Protein concentrations were quantified using a Micro BCA^TM^ Protein Assay Kit (Thermo Fisher Scientific, Rockford, IL, USA).

### Nanoparticle Tracking Analysis (NTA)

To examine the size distribution and more accurately determine particle concentrations, the Nanosight NS500 platform (Malvern Panalytical, Malvern, Worcestershire UK) for NTA was used following EV isolation. Samples were diluted with exosome buffer, loaded onto the Nanosight, and analysis settings were optimised and held constant between samples. Mean, mode, median and particle concentration were estimated by their Brownian motion which was analyzed based on 3 videos of 30 seconds each [17].

### Statistical analysis

Statistical significance between two groups was determined using unpaired Student t-tests, corrected for multiple comparisons using the Holm-Sidak method. Brown-Forsythe and Welch ANOVA tests, with Games-Howell’s correction for multiple comparisons, or, 2-way ANOVA with multiple comparisons by uncorrected Fisher LSD’s Test were used when comparing multiple groups. P-values lower than 0.05 were considered significant; * indicates p ≤ 0.05, ** indicates p ≤ 0.01, and *** indicates p ≤ 0.001.

## Results

### Phleboviral particles were observed in Leishmania-infected sandfly midguts

While examining sections of the sandfly midgut by TEM, we observed what appeared to be virus-like entities within intracellular compartments of midgut cells (S2 Fig).

A brief study of the literature informed us that phlebotomine sandflies of the genus Lutzomyia in the New World and Phlebotomus in the Old World are the common vectors of *Leishmania* and various Phleboviruses [9, 18]. To confirm virus presence in the midgut contents, we prepared samples of sandfly gut lavages for proteomic analysis. Importantly, we took samples from sandflies both infected and uninfected with *Leishmania*, as this would indicate whether the presence of the virus was *Leishmania*-dependent.

Results from mass spectrometry analysis revealed that peptide sequences were analogous to proteins of the *Bunyavirudae* family of viruses (S1 Table), at the moment of analysis, phleboviruses were classified under the family *Bunyaviridae* instead of *Phenuivirdiae* [10]. While this was insufficient for precise identification of viral species, it confirmed that viral entities were present in the sandfly midgut. These findings lead to the possibility of their regurgitation alongside *Leishmania* parasites during the sandfly bloodmeal.

### SFSV—L. major co-infection exacerbates cutaneous leishmaniasis

Mass spectrometry analysis confirmed that the particles observed in TEM were viral and indicated that these virus particles were being released from the midgut cells into the lumen of the midgut, however, the implications of this observation were unclear. As *Leishmania* species and phleboviruses share the same sandfly vector, we wanted to investigate the effect a co-infection could have on CL severity and progression. Thus, wild-type C57BL/6 mice were infected with either *L*. *major* (n = 6) or *L*. *major* and SFSV (n = 8), and monitored for 15 weeks.

**Fig 1:**
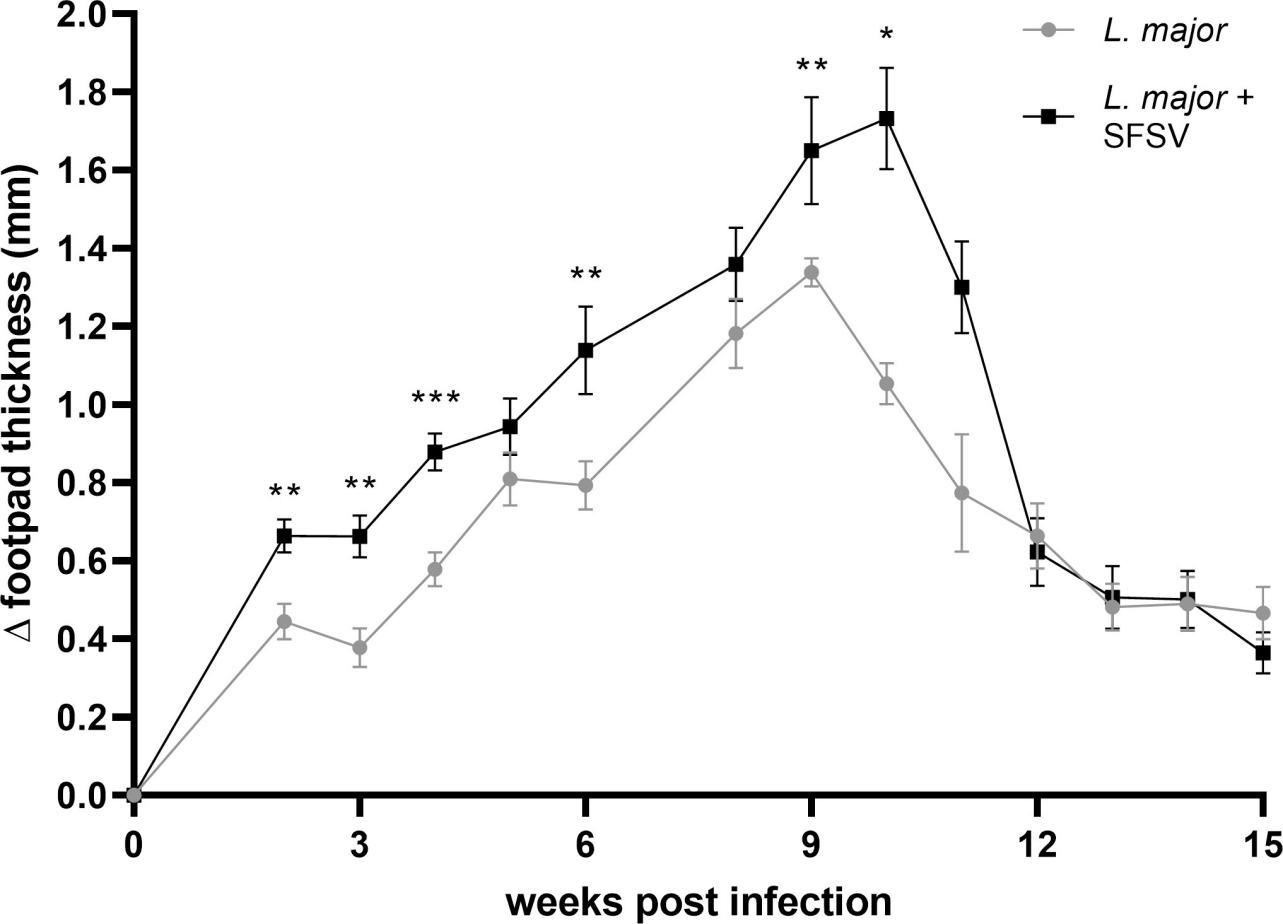
Footpad swelling in *L*. *major*- and *L*. *major*-SFSV co-infected mice. Wild-type C57BL/6 mice were (co-)infected with *L*. *major* (n = 6) or *L*. *major* and SFSV (n = 8). Changes in footpad thickness were monitored for 15 weeks. For the inset graph, P-values were calculated by student’s t-test. For CL progression and footpad swelling, 2-way ANOVA with multiple comparisons by uncorrected Fisher LSD`s Test was used. * p ≤ 0.05, ** p ≤ 0.01, and *** p ≤ 0.001.

Remarkably, SFSV and *L*. *major* co-infection in wild-type groups exacerbated CL, as demonstrated by the significantly greater swelling of the co-infected footpads. This effect was sustained from week 1 up until 11 weeks post-infection (Fig 1), then resolved in both mouse groups by the end of the 15-week period. This exacerbation supported our hypothesis that viral presence may influence clinical manifestations of CL.

### SFSV induces IRF3 and MAP kinase signaling pathways and NF-ĸB translocation

Next, we attempted to elucidate the mechanisms involved in this exacerbated pathology. SFSV is a single strand RNA virus, so we first investigated signaling pathways known to be triggered by this ligand.

By western blot, we observed that the virus induced a pro-inflammatory state at early time points after SFSV infection. We noted transient upregulation of MAP kinase p38 (important in anti-pathogen responses), ERK1/2 phosphorylation, and nuclear translocation of NF-ĸB (S3 Fig), an important transcription factor heavily involved in host response to pathogens, among other activities [19]. Upregulated phosphorylation of the IRF3 pathway was also observed, as demonstrated by increased phospho-TBK and phospho-IRF3.

### SFSV-induced exacerbation of cutaneous leishmaniasis is TLR3-dependent

**Fig 2:**
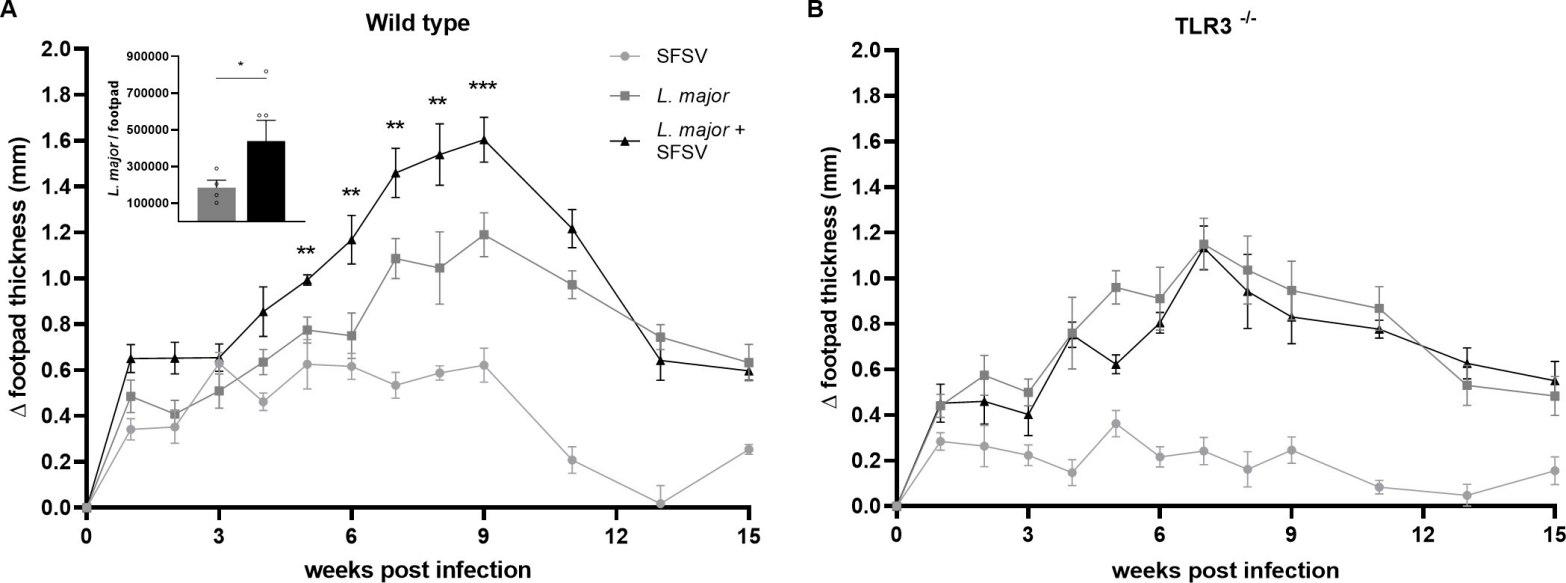
In TLR3^-/-^ mice, SFSV-induced exacerbation of CL inflammation is diminished. **A)** Wild-type mice were infected with *L*. *major* (n = 8) or co-infected (n = 9). Changes in footpad thickness were monitored for 15 weeks. Footpads were harvested and parasite load was measured. **B**) T*LR3*^*-/-*^ mice were *L*. major-infected (n = 8) or *L*. *major* and SFSV co-infected (n = 9). Footpad swelling was also monitored for 15 weeks. All p-values were calculated by 2-way ANOVA with multiple comparisons by uncorrected Fisher LSD`s Test. * p ≤ 0.05, ** p ≤ 0.01, and *** p ≤ 0.001.

As we established that SFSV alone has a pro-inflammatory effect, we wanted to further examine its effect *in vivo*. We next infected groups of wild-type C57BL/6 mice with either *L*. *major* alone (n = 8) or *L*. *major* and SFSV (n = 9) to determine if the presence of SFSV impacted parasite load. We monitored the mice over 8 weeks, at which time co-infected footpads again demonstrated visibly more redness and swelling than footpads infected with *L*. *major* alone (Fig 2A). Mice were sacrificed, their footpads harvested, and parasite burden per footpad was determined by limiting dilution assay.

Mice co-infected with SFSV and *L*. *major* displayed significantly greater parasite burden than mice infected with *L*. *major* alone (as calculated by Student’s t-test; p = 0.0159) (Fig 2A). The mean number of *L*. *major* per footpad was 467,200 in the co-infected group, compared to 136,000 in the parasite-infected group; more than a three-fold increase in parasite load.

Now that viral presence was demonstrated to influence CL pathology, we wanted to further elucidate the involved signaling pathways *in vivo*. *Leishmania* is known to induce macrophage dysfunction through direct manipulation of common macrophage signaling pathways [20, 21]. It has been documented that endosomal TLRs are critical to the mediation of *L*. *major*-induced CL [22]. Additionally, although phleboviruses have ssRNA genomes, in the course of their replication they generate dsRNA intermediates [23]. The presence of dsRNA would signal that SFSV was present and actively replicating. We therefore decided to examine the role of TLR3, a dsRNA PRR, in SFSV and co-infection-induced CL. Groups of 8 or 9 wild-type and *TLR3*^-/-^ mice on a C57BL/6 background were infected with either SFSV and *L*. *major* or *L*. *major* alone, with a control group solely infected with SFSV. Infected mice were monitored for 15 weeks.

Self-healing CL had a similar duration in both genotypes, regardless of SFSV co-infection. Wild-type mice (Fig 2A) co-infected with SFSV and *L*. *major* experienced significantly more severe CL than mice infected with *L*. *major* alone. However, no difference in disease severity between infection conditions was observed in *TLR3*^*-/-*^ mice (Fig 2B). This demonstrated that TLR3 is involved in the exacerbation of CL caused by SFSV co-infection. Furthermore, *TLR3*^-/-^ mice infected with SFSV alone experienced less footpad inflammation than SFSV-infected wild-type mice, further supporting the idea that SFSV can modulate CL in a TLR3-dependent manner. This raises the possibility that the increased severity of CL associated with SFSV-*L*. *major* co-infection may be triggered by TLR3-initiated pathways.

### IFNAR may play a role in the MAVS-mediated SFSV-induced exacerbation of cutaneous leishmaniasis

Next we wanted to look to another dsRNA-sensing PRR family, the cytoplasmic RIG-I family [24, 25], situated directly downstream from the mitochondria-associated MAVS adaptor proteins. Once RIG-I binds its ligand, dsRNA, it translocates to the mitochondria where it recruits and activates MAVS, which continues activation of the signal transduction pathway initiated by RIG-I [26]. As a critical viral PRR adaptor protein, we were interested in the possible role of MAVS, and RIG-I by association, in the hyperinflammation caused by SFSV and *L*. *major* co-infection. To this end, we infected wild-type and *MAVS*^-/-^ mice on a C57BL/6 background with *L*. *major* or SFSV alone, or co-infected with SFSV and *L*. *major*. As before, we followed the experiment for 15 weeks.

**Fig 3:**
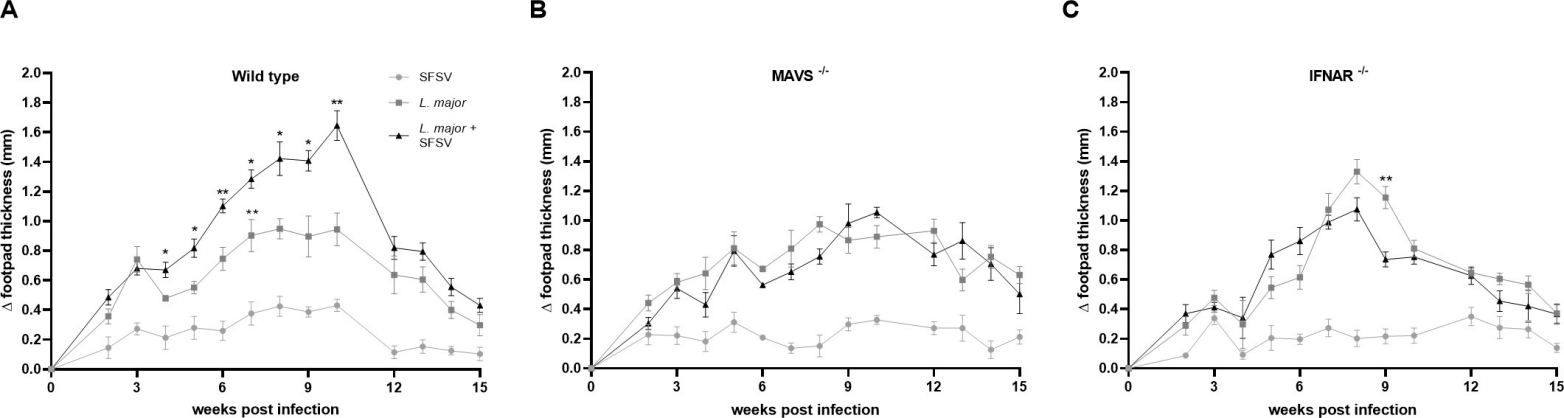
MAVS^-/-^ mice display no SFSV-induced exacerbation of CL inflammation. **A**) In the wild-type control group, mice were infected *L*. *major* (n = 7), SFSV (n = 6) or both pathogens (n = 9). **B, C**) In all MAVS^-/-^ and IFNAR^-/-^ mice groups, 5 and 6 mice were infected, respectively. In all groups, CL exacerbation was monitored by measuring footpad swelling over 15 weeks. 2-way ANOVA with multiple comparisons by uncorrected Fisher LSD`s Test was used to calculate all p-values. * p ≤ 0.05, ** p ≤ 0.01, and *** p ≤ 0.001.

Once more, we observed prolonged and significantly more severe CL in wild-type mice infected with both *L*. *major* and SFSV compared to those infected with *L*. *major* only (Fig 3A). However, in the *MAVS*^-/-^ mice, CL caused by either *L*. *major* infection or *L*. *major*-SFSV co-infection resulted in similar levels of inflammation (Fig 3B), implicating MAVS in the increased CL severity caused by SFSV. Evidently, both endosomal and cytoplasmic viral PRRs can mediate the exacerbation of CL resulting from SFSV and *L*. *major* co-infection. Interestingly, SFSV infection by itself did not show significant modification in the swelling triggered between groups, suggesting that TLR3 may be the main driver behind the viral co-infection’s enhanced CL pathology.

Next, we investigated viral recognition signaling further downstream than PRRs to ascertain how these receptors were mediating the observed increase in CL severity. Type I interferons are products of both TLR3 and RIG-I signaling pathways [24]. Their subsequent binding to IFNAR results in the induction of an antiviral state within host cells. To determine the role of a key antiviral receptor in phlebovirus-driven hyperinflammatory CL, we infected groups of wild-type and *IFNAR*^-/-^ mice with *L*. *major*, *L*. *major* and SFSV, and SFSV alone. We then monitored mice for 15 weeks.

In *IFNAR*^-/-^ mice, there was no prolonged increase in CL severity in co-infected mice compared to *L*. *major* infected mice (Fig 3C); interestingly, CL was more severe in *L*. major-infected groups compared to wild-type controls, which suggested IFNAR plays a factor in the SFSV-induced CL enhancement.

### SFSV-L. major co-infection leads to an increased recruitment of leukocytes to the site of infection

We then examined whether SFSV-aggravated CL could be attributed to an elevated initial infection of inflammatory myeloid cells. Wild-type C57BL/6 mice were infected with PBS (n = 7), *L*. *major* (n = 8) or SFSV (n = 7), or co-infected with *L*. *major* and SFSV (n = 9) in the peritoneal cavity, a convenient technique to obtain and investigate large volumes of inflammatory cells. We thus collected peritoneal cavity lavages 6 hours post-infection, as previous research in our lab has demonstrated that leukocyte recruitment in response to *L*. *major* infection reaches a maximal peak at 6 hours, after which it declines over a 48 hour period [27].

**Fig 4:**
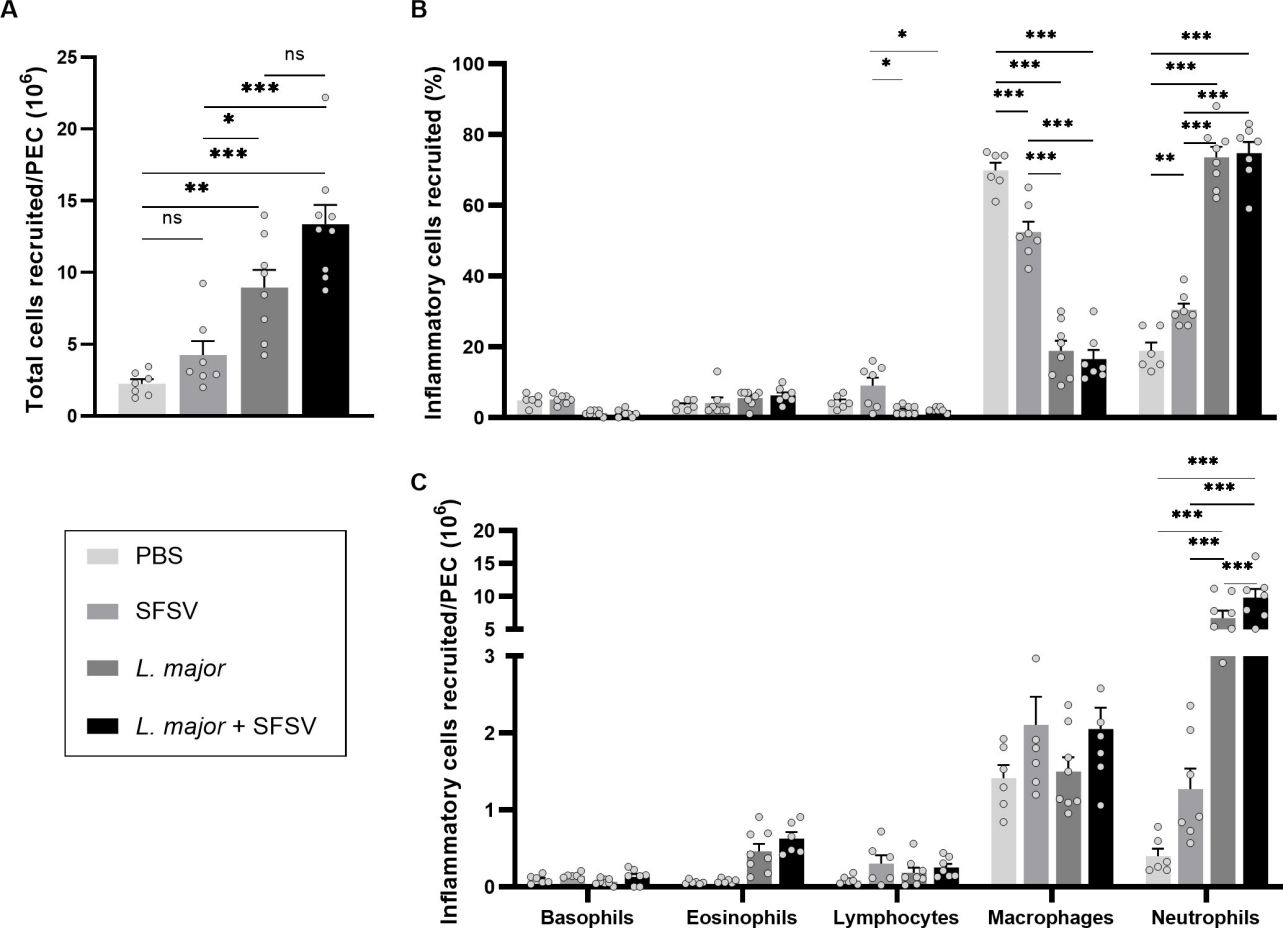
The effect of L. major and SFSV co-infection on leukocyte recruitment to the site of infection. Wild-type C57BL/6 mice were inoculated with PBS (n = 7), SFSV (n = 7), or L. major (n = 8), or co-infected with L. major and SFSV (n = 9). Peritoneal cavity lavages (PEC) were collected and recruited cells were inspected by microscopy. **A)** The total number of recruited leukocytes per PEC was calculated. **B)** Additionally, the different cell types were counted and their percentage of occurrence was calculated based on the total number of leukocytes recruited. **C)** Lastly, we calculated the total number of cells recruited for each cell type, based on the total number of leukocytes recruited to the peritoneal cavity. P-values were calculated by 2-way ANOVA tests with Tukey’s correction for multiple comparisons, Bars = Mean ± SEM, * p ≤ 0.05, ** p ≤ 0.01, and *** p ≤ 0.001? PEC = peritoneal cavity.

As expected, the different pathogens induced leukocyte recruitment with varying magnitudes. *L*. *major* inoculation, with or without SFSV, initiated a significantly stronger response than SFSV alone (Fig 4A). Interestingly, a small increased influx was observed after co-infection compared to *L*. *major* infection, however this was not significant.

We further wanted to investigate the cellular distribution between SFSV (n = 7), *L*. *major* (n = 8) or co-infected (n = 7) groups and the controls (n = 6). Among the different cell types present 6 hours post-infection, macrophages were most abundant in PBS (70%) and SFSV (53%) peritoneally inoculated mice (Fig 4B). Upon parasitic (co-)infection the largest population shifted towards neutrophils; 73% and 75% of the inflammatory cells recruited in response to *L*. *major*- or *L*. *major* and SFSV-infection were neutrophils, while macrophages accounted for merely 19% and 17%, respectively.

When examining the absolute numbers of inflammatory cell types recruited to the peritoneal cavity, it was clear that co-infection indeed induced an increased influx of leukocytes. Supporting earlier findings, the main population of inflammatory cells present consisted of neutrophils [27, 28]. Additionally, we observed that co-inoculation initiated the largest neutrophil influx compared to controls; a 25-fold increase in co-infected mice versus 17-fold in *L*. *major*-inoculated groups (Fig 4C). Small numbers of eosinophils were also present in all groups without significant differences. Nevertheless, we noted that *L*. *major* or *L*. *major* and SFSV co-infection led to elevated eosinophil numbers.

### SFSV co-infection increases the ability of L. major to infect host cells

To investigate whether SFSV presence might influence *L*. *major* infectivity, we examined macrophages and neutrophils, the known host cells of *Leishmania* [29]. Percentage of infected cells and number of amastigotes were counted and compared between *L*. *major* and co-infected groups (n = 7).

**Fig 5:**
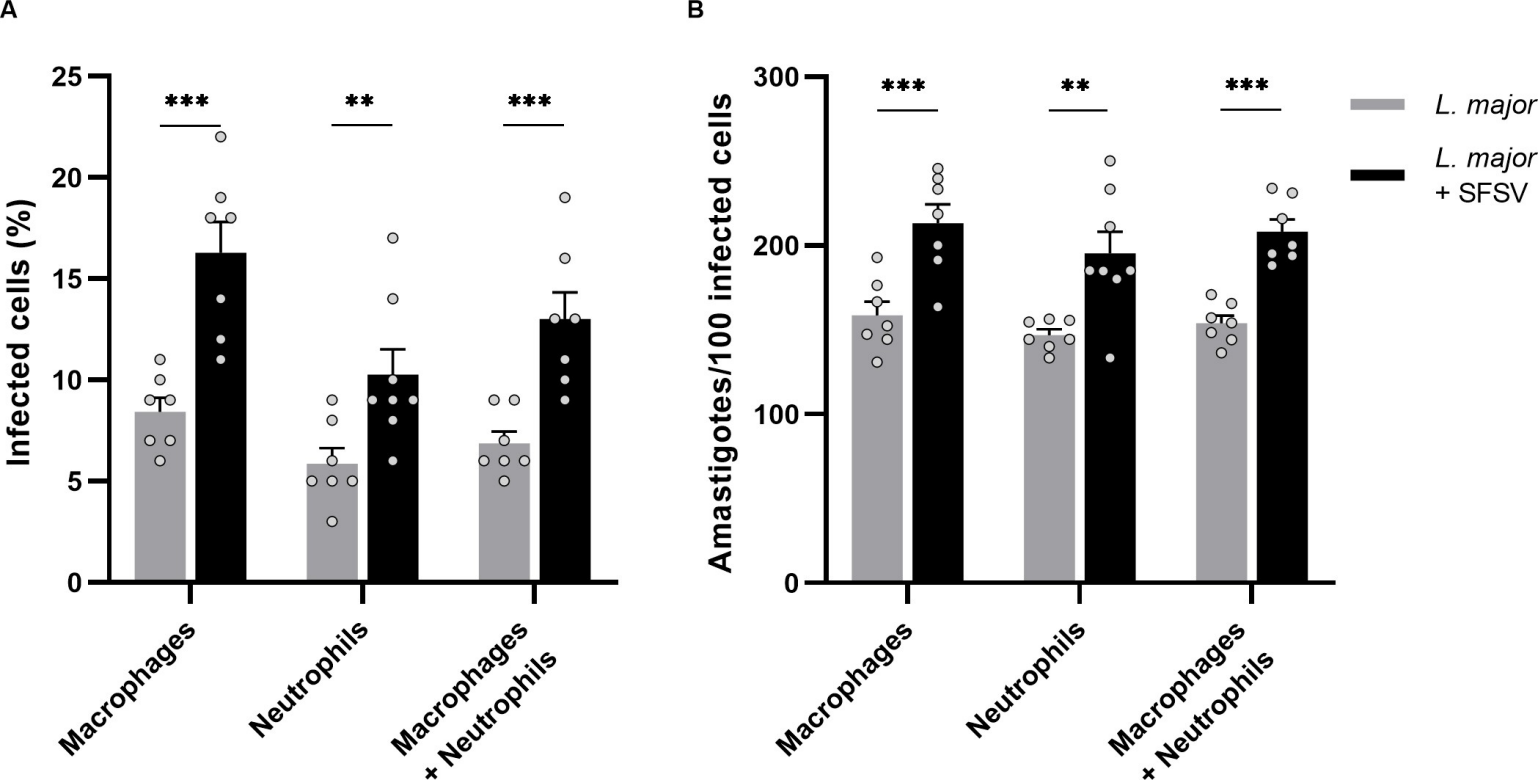
The effect of SFSV co-infection on infected host cells. Mice were infected with L. major alone (n = 7) or co-infected with SFSV (n = 7). Macrophages and neutrophils were inspected and **A)** the percentage of infected cells was calculated. Additionally, **B)** the number of amastigotes per 100 cells was determined. All p-values were calculated by unpaired Student t-tests with Holm-Sidak correction for multiple comparisons, Bars = Mean ± SEM, * p ≤ 0.05, ** p ≤ 0.01, and *** p ≤ 0.001.

Remarkably, we found that upon viral co-infection, infectiveness was elevated; amastigotes were present in ~8.5% of macrophages when inoculated with *L*. *major* alone, compared to ~16.5% when co-infected. A similar trend was visible in neutrophils, where the affected cells increased from ~6% to ~10%. Overall, co-infection led to a ~2-fold (p = 0.0024) increase of *Leishmania*-infected inflammatory cells (Fig 5A). This trend was maintained when looking at the total number of amastigotes residing in infected leukocytes. Upon co-inoculation, approximately 55 and 48 more parasites infected macrophages and neutrophils, respectively. The overall number of amastigotes present was increased 1,3-fold (p = 0.0305) when infected with both *L*. *major* and SFSV (as calculated by Student’s t-test; p) (Fig 5B). Viral presence seemingly influences *L*. *major* infectiveness.

### Parasitic inoculation modulates cytokine and chemokine profiles

We then examined whether this differentially induced cell recruitment could be associated with an accumulation of inflammatory mediators. Cytokine and chemokine profiles 6 hours post-infection were calculated through multiplex arrays and PBS (n = 7), *L*. *major* (n = 7) or SFSV (n = 6) alone, or *L*. *major* and SFSV-inoculated (n = 9) groups were compared.

**Fig 6:**
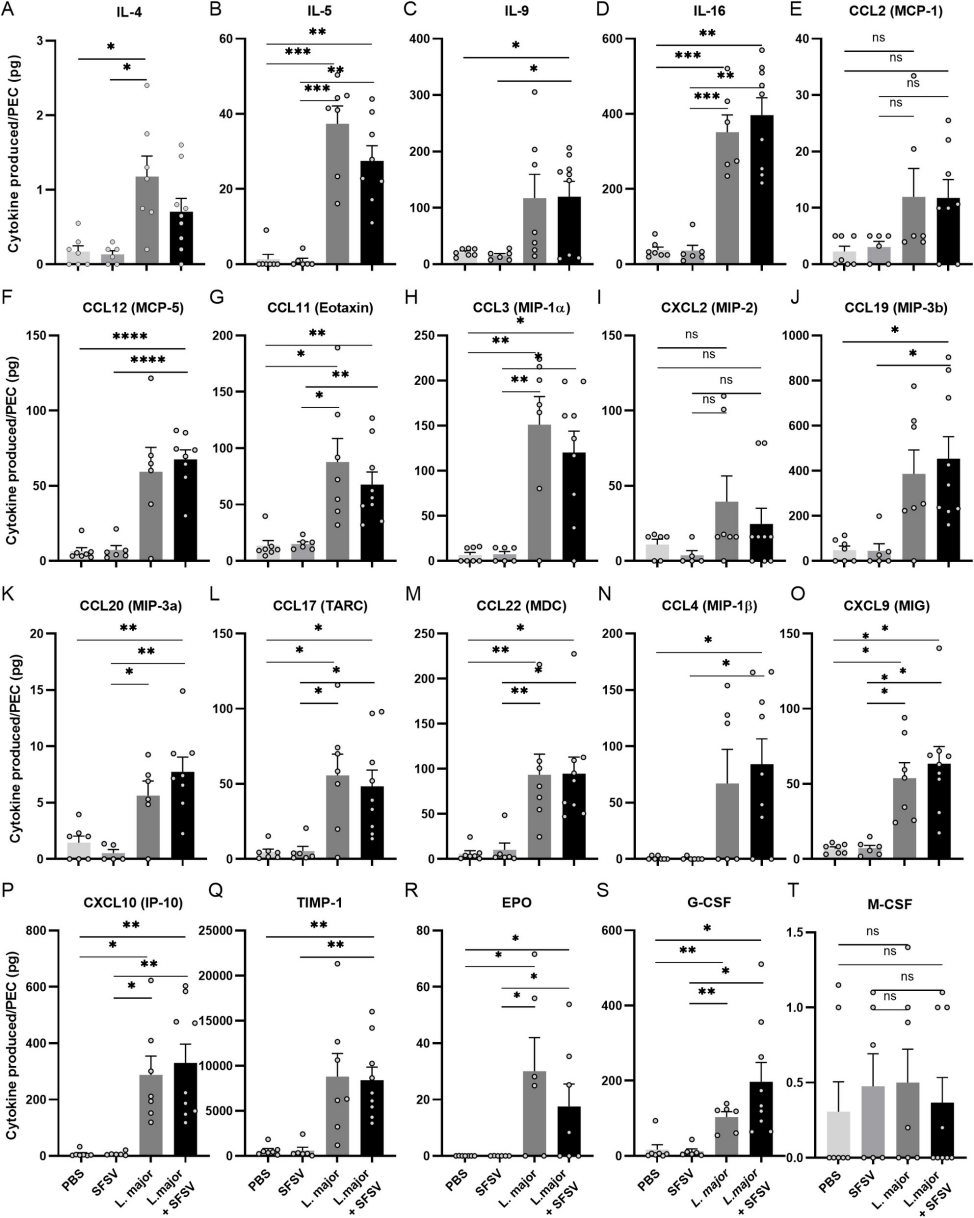
Pathogen-induced cytokine and chemokine profiles. Modulation pattern in cytokine and chemokine release in the peritoneal cavity 6 hours post-infection for (A-D) cytokines (E-I), chemokines affecting leukocytes of the innate immune system (J-P), lymphocyte chemoattractant molecules, and (Q-T) other immunomodulatory molecules. All p-values were calculated by Brown-Forsythe and Welch ANOVA tests with Games-Howell’s correction for multiple comparisons, Bars = Mean ± SEM, * p ≤ 0.05, ** p ≤ 0.01, and *** p ≤ 0.001. PEC = peritoneal cavity.

IL-4, known to counteract *Leishmania* clearance by initiating macrophage alternative activation [30], was upregulated, with *L*. *major*-alone infected groups displaying the largest increase, albeit in low concentrations (Fig 6). Interestingly, we established a similar trend for IL-5. The increased eosinophil recruitment previously observed may be influenced by this finding, as IL-5 enhances the differentiation and proliferation of eosinophil progenitors in the bone marrow and their migration to the infection site [31]. IL-9 levels, mainly produced by and acting on mast cells [31, 32], were unexpectedly elevated. That said, this modulation might be involved through its additional role in Th2 cell expansion, essential for parasite survival [33, 34]. Lastly, IL-16 was strongly induced in *L*. *major* (co-)infections. No significant differences were detected between *L*. *major* infected or *L*. *major* and SFSV co-infected groups, although co-infection resulted in a slight elevation. In SFSV-alone infected groups, cytokine expression was not induced (Fig 6A–6D).

We observed similar trends for innate inflammatory chemokines; while in SFSV-infected mice chemokine levels were comparable to the control groups, *L*. *major*-(co-)infection demonstrated modulation. Macrophage attractant molecules CLL2 and CCL12 levels, for example, were increased. Both are mainly produced by monocytes and macrophages and are primarily involved in the migration and infiltration of monocytes [33, 35, 36]. The clear upregulation of CCL2, CCL12 and CCL11 after parasitic infection implies an additional importance in eosinophil mobilisation [35, 36]. Surprisingly, only two chemokines involving neutrophil recruitment were modulated after *L*. *major* (co-)infection: a clear increase was detected for CCL3 and CXCL2, although statistically not significant (Fig 6E–6I).

It is well-known that the type of immune response mounted by the host is determinant for *Leishmania* survival [37]. We thus wanted to examine the chemokines of the adaptive immune system in response to infection. Consistent with our previous observations, modulation was only detected when *L*. *major* (co-)infected. Chemokines involved in general lymphocyte recruitment were upregulated, including CCL19 and CCL20. CCL17 and CCL22, more specifically responsible for Th2 lymphocyte recruitment, showed similar modulation. Regulatory CD4^+^CD25^+^ lymphocytes are determinant for the final host response by inhibiting CD4^+^CD25^+^T cells from producing a Type 1 response [37]. Both CCL4, the main regulator of CD4^+^CD25^+^ lymphocyte recruitment, and CXCL9 and CXCL2, responsible for a Th1 response, had elevated levels in *L*. *major* alone or SFSV-co-infected mice (Fig 6J–6P).

Lastly, we examined the various immunomodulatory proteins released at the site of infection. A clear upregulation was observed for Tissue inhibitor metalloproteinase-1 (TIMP-1) in parasite-infected groups. This protein was first described for its inhibitory effect on matrix metalloproteinases (MMPs), but has additional cytokine-like functions through induction of cell signaling. TIMP-1 is also involved in hematopoiesis, through which it can further regulate inflammatory events [38–40]. For erythropoietin (EPO), we also detected alterations when *L*. *major* (co-)infected, with the biggest increase in mice inoculated with *L*. *major* alone. Granulocyte Colony-Stimulating Factor (G-CSF), with its role in the maturation and activation of granulocytes, including neutrophils, was elevated after parasitic infection, given the increased neutrophil recruitment. As analyzed previously, macrophage mobilisation was not significantly modulated upon infection with the different pathogens, as reflected in the release of M-CSF, a hematopoietic regulator of the monocytic cell lineage [41], which was not upregulated after infection with SFSV, *L*. *major*, or after co-infection (Fig 6Q–6T).

### Leishmania-released exosome levels were increased after viral co-infection

**Fig 7:**
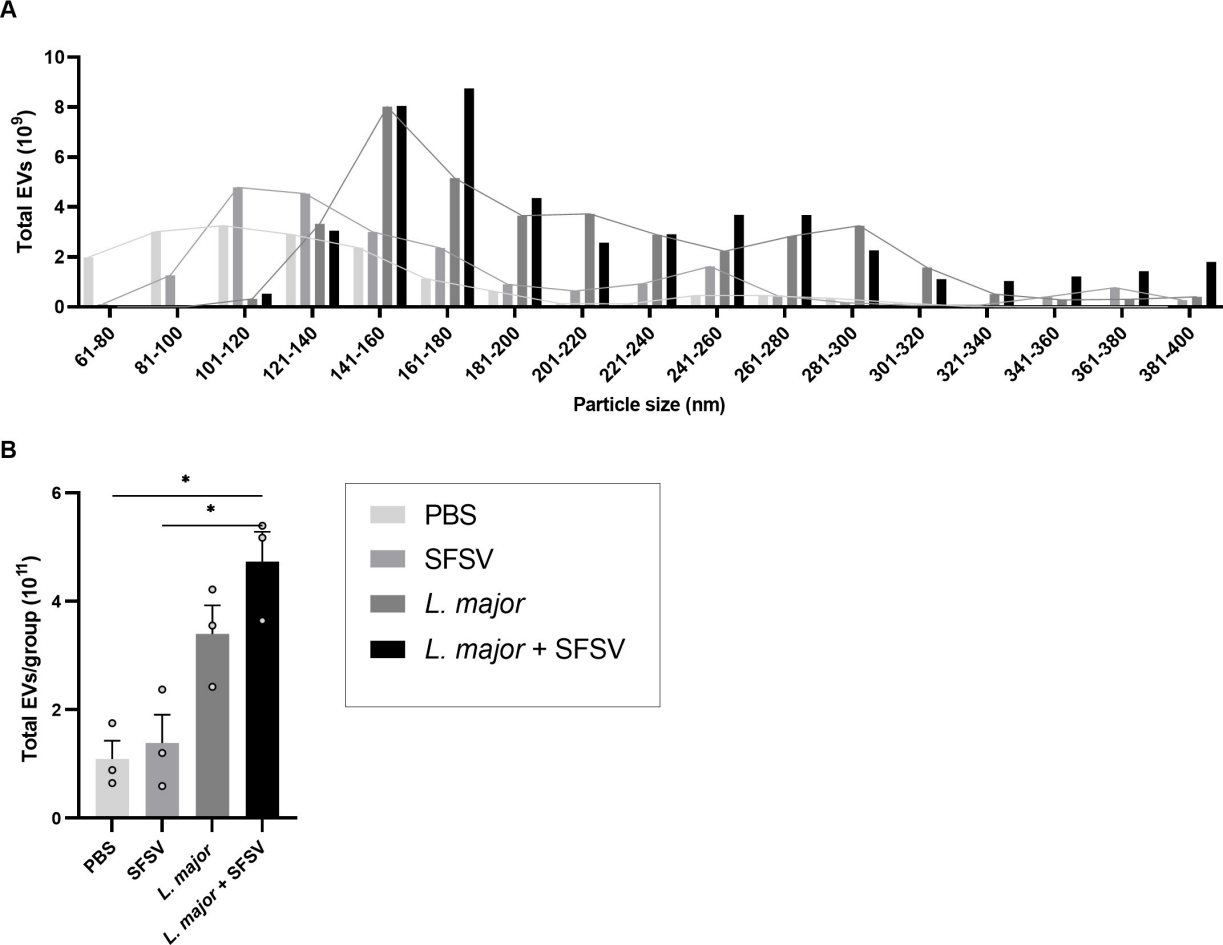
The effect of *L*. *major* (co-)infection on the number and size distribution of EVs released in the peritoneal cavity 6 hours post-infection. For all groups (n = 3, each representing a pool of 3 mice), extracellular vesicles were isolated and population homogeneity and particle size distribution were analyzed through NTA. **A)** The size distribution of the total number of EVs isolated was determined and **B)** the total number of EVs isolated from the peritoneal cavity lavages were calculated and compared. **B)** All p-values were calculated by Brown-Forsythe and Welch ANOVA tests with Games-Howell’s correction for multiple comparisons, Bars = Mean ± SEM, * p ≤ 0.05, ** p ≤ 0.01, and *** p ≤ 0.001. PEC = peritoneal cavity.

As we have previously established that co-egested *Leishmania* exosomes exacerbate CL skin lesions [7], we were interested in whether phleboviral presence could modulate exosome concentration. Isolated EVs were therefore examined by TEM imaging for identification and morphological investigation. TEM images showed vesicles with the characteristic bi-layer membranes and sizes ranging from 40–120 nm; exosome presence in the preparations was thus confirmed (S4 Fig). Following isolation, population homogeneity and particle size distribution were analyzed through NTA. We found that the preparations contained EVs with sizes ranging between 60 and 900nm. The distribution additionally suggested that peak concentrations consisted of vesicles with a diameter between 100–200nm (S4 Fig). We then calculated the size distribution of the total number of EVs isolated (n = 3, each representing a pool of 3 mice). A change was detected upon parasitic infection; while the main distribution in control mice consisted of particles with a diameter of ~80-180nm, SFSV inoculation initiated vesicle release with sizes ranging up to ~260nm. *L*. *major* infection, on the other hand, shifted size distributions to a range of ~80 to 400nm. We did not detect differences in populations between *L*. *major*- or co-infected mice (Figs 7A and S4). Lastly, the total number of isolated EVs were calculated (n = 3, each representing a pool of 3 mice). Here, it was clear that SFSV infection alone did not induce EV release. Parasitic infection, on the other hand, showed a clear increase in total EVs present (Fig 7B). Moreover, the largest number was found in co-infected mice. Considering that almost all eukaryotic cells release EVs, this elevation reflects the increased inflammatory cell recruitment. It could furthermore suggest a modulatory role of SFSV on *L*. *major* exosomes. Overall, it can be concluded that parasitic infection, especially co-infection, induced larger sized EVs. The number of total EVs isolated from the peritoneal cavity lavages was additionally increased, reflecting the elevated numbers of migratory inflammatory cells.

## Discussion

The leishmaniases, a group of chronic infectious diseases caused by etiologic agents of one genus [1, 2], are characterized by extraordinarily diverse pathologies. Cutaneous leishmaniasis itself is a disease with wide-ranging severity and associated complications. The factors that are currently known to determine its severity are mainly host- or parasite-derived [3].

Our observation of phlebovirus-like particles in the sandfly midgut led us to hypothesize that phleboviruses could be co-egested along with *Leishmania*, and could result in a modulation of CL. Our results demonstrated that wild-type mice co-infected with *L*. *major* and SFSV resulted in a notably exacerbated CL, and SFSV and *L*. *major* co-infected footpads had an approximately three-fold larger parasite load than mice infected with *L*. *major* alone. Evidently, the presence of the virus at the infection site facilitated parasite infectivity, and perhaps a stronger host inflammatory response, but the exact mechanism by which this occurs remains unclear. There has been an emerging interest in *Leishmania* and viral co-infection. It has, for example, been established that the severity of murine mucocutaneous leishmaniasis (MCL), caused by *L*. *(V*.*) guyanensis*, is in fact mediated by *Leishmania* RNA Virus 1 (LRV1), a virus infecting the parasite [14]. The nature of the ecological relationship between SFSV and *L*. *major* differs from that of LRV1 and *L*. *(V*.*) guyanensis*, but the exacerbated disease is a similar outcome, and illustrates another factor modulating CL severity. Interestingly, Rath and coworkers recently shed light on the possible influence of *L*. *amazonensis*, a parasite of the Amazon, and rodent-borne phlebovirus Icoaraci co-infection; they demonstrated an aggravated infection index *in vitro* and suggested the importance of the PKR and IFN1 axis [42].

In an attempt to elucidate the mechanisms through which SFSV works, we looked at pathways triggered by ssRNA. We observed that early after infection, the virus induced a pro-inflammatory state, *in vitro*. Phosphorylation of IRF3 was found to be upregulated, and important transcription factor that can be activated to allow translated of type I interferons and other cytokines. Contradictory to our findings, SFSV has previously been reported to block IRF3 through the NSs protein and thereby suppressing the induction of type I IFNs [43]. However, it has been demonstrated by others, as well as us, that IRF3 was modulated in the context of viral co-infection at the initial moments of infection [42, 44].

Previously, Gowen and coworkers reported that during infection by Punta Toro Virus, another phlebovirus, host TLR3-mediated immunopathology controls mouse morbidity and mortality [45]. Many others have proposed that the lesions that typify CL are actually the result of host defense mechanisms gone too far [46–48], as opposed to damage directly perpetrated by the parasites themselves. Attempting to determine a more specific mechanism by which SFSV modulates CL, we emulated the aforementioned study by Gowen and researchers and utilized TLR3^-/-^ mice and wild-type controls for our subsequent experiment. In TLR3^-/-^ mice, no difference in CL severity was observed between *L*. *major* infections and SFSV and *L*. *major* co-infection, whereas SFSV co-infection exacerbated inflammation in wild-type mice. Evidently, the addition of SFSV to *L*. major infection activates TLR3 signaling to a greater extent than *L*. *major* alone, resulting in an intensified CL that could possibly be attributed to an immunopathological response triggered by the recognition of SFSV RNA. This seems reasonable given that Toll-like receptors are critical to the recognition of viral genetic material [49, 50]. In the absence of TLR3, hosts are still able to clear CL caused by *L*. *major* and SFSV co-infection, in a non-prolonged time, and CL severity is lessened.

Similar CL outcomes were also observed in MAVS^-/-^ mice co-infected with SFSV and *L*. *major* and *L*. *major* alone. This finding supports that it is the host response to the parasite-SFSV co-infection that spurs a more aggressive CL progression. A paper published by the Vidal lab [24] reported that RIG-I, the PRR directly upstream from MAVS and responsible for its activation, as well as TLR3, responded to infection by another RNA virus, influenza A virus, through type 1 interferon signaling, resulting in a positive feedback loop. Additionally, the importance of type 1 IFN pathways in phleboviral co-infection with *L*. *amazonensis* was demonstrated by a reduced infection index in murine peritoneal macrophages after phleboviral and *L*. *amazonensis* co-infection [42]. This corroborates our findings that the hyperinflammatory response due to SFSV and *L*. *major* co-infection could be dependent on both MAVS and TLR3, and further strengthens the idea that it is a host-mediated immunopathological response to the virus that may ultimately be causing CL aggravation. Although, in IFNAR^-/-^ mice, no prolonged increase in CL severity was observed between (co-)infected groups, mice infected with *L*. *major* alone displayed greater footpad swelling that in wild-type mice. Further studies are needed in order to determine exactly how IFNAR figures into SFSV-induced exacerbation of CL, although it appears to play a modulatory role in CL overall. Rossi et al. described a worsened outcome of murine leishmaniasis when co-infected with Toscana phlebovirus (TOSV), a virus very similar to SFSV and member of the phleboviruses. They furthermore demonstrated that viral co-infection with LCMV induced the down-regulation of the IFN-γ receptor in a type I IFN-dependent manner, proposing a role for IFNAR in viral co-infection [44]. Further studies of interferon production throughout the course of CL would help determine the role of IFNAR in the context of phlebovirus and *L*. *major* co-infection.

Having established a clear modulatory role of SFSV in the clinical manifestation, we wanted to identify innate inflammatory events that could lead to the development of this hyper-inflammation. To our knowledge, this is the first reported demonstration that viral co-infection can alter inflammatory cell recruitment; our results demonstrated that *L*. *major* and SFSV co-infection increased inflammatory cell numbers at the site of infection, supporting the previously observed exacerbated footpad swelling. As has been extensively described before [27, 28], neutrophils dominated the cell population. Previous *in vitro* research with *L*. *amazonensis* and phlebovirus co-infected murine peritoneal macrophages displayed increased infection indexes without higher parasite numbers [42]. Our *in vivo* results supported these findings with a doubled percentage of infected macrophages and neutrophils, the known hosts of *Leishmania*. Interestingly, this further suggests modulatory properties of SFSV on the host immune system; as we observed increased parasite load after co-infection *in vivo*, viral presence likely impacts the infectivity of *L*. *major* through an as-yet-unknown mechanism.

Extensive research regarding cytokine and chemokine release upon phleboviral infection is lacking. However, *in vivo* research in C57BL/6 mice with Severe Fever Thrombocytopenia Syndrome Virus (SFTSV), genus *Phlebovirus*, reported a cytokine storm with peak concentrations 24 hours post-infection [51]. Additional *in vitro* studies performed on bone marrow derived macrophages (BMDM) with the highly pathogenic RVFV showed that cytokine and chemokine production is highly dependent on the NSs protein [52]. Our results demonstrated that a wide array of cytokines and chemokines were modulated upon parasitic infection, with or without SFSV. To our surprise the virus alone did not modulate inflammatory protein levels. It would be interesting to look at mediator levels at different time points post-infection, as it is possible that 6 hours is too early to detect any modulation by the virus. We were furthermore unable to detect significant differences between *L*. *major* alone or co-infected mice. We can therefore make no assumptions regarding the modulatory role of SFSV on cytokine and chemokine release. Notwithstanding, it is tempting to suggest that the aggravated CL is mainly caused by increased recruited leukocytes rather than the induction of a cytokine storm. This was further supported by elevated EV numbers, released by the majority of eukaryotic cells and protozoan parasites, including *Leishmania*. Future analysis of the protein content in these EVs may better characterize the mechanics behind this elevation. It is also possible that viral presence might favour parasite entrance into the cell, as suggested by the increased number of parasites in infected macrophages and neutrophils, but does not progress to a stronger inflammation (based on chemokines and cytokines).

Ultimately, our work represents an advancement in the understanding of the mechanisms controlling CL pathology. Here, we determined that SFSV co-infection with *L*. *major* results in a greater parasite load in infected mice, which manifests through an exacerbated form of CL; ostensibly, this aggravated CL is the result of a host immunopathological response that occurs through TLR3- and MAVS-dependent pathways. With respect to the increased inflammation observed in SFSV-*L*. *major* co-infections, the increased parasite burden appears to be secondary to an immunopathological response initiated by PRR recognition of SFSV RNA. We furthermore established that innate immunological events were additionally altered; SFSV presence increases initial recruitment of inflammatory cells and parasite infectivity. *Leishmania* exosome numbers are also increased after co-infection, reflecting the elevated leukocyte numbers. This modulation is, however, not paralleled by inflammatory mediators released at the site of infection. That said, further studies of cytokine expression throughout the course of CL progression might permit a more complete mechanistic understanding of how exactly this co-infection, with its greater parasite burden, provokes a more intense immune response. Additionally, repeating this study with another phlebovirus, distinct from SFSV, could shed more light on the exact mechanisms and specificity of our observations. Another interesting objective would be to compare the infection rate between co-infected mice, and mice firstly infected with *L*. *major* and, at a later time point, superinfected with SFSV. Possible vector-related mechanism could be revealed. It is furthermore plausible that in nature, an individual suffering from *Leishmania*-infection is later on superinfected with another virus, such as phleboviruses.

Better characterization of the factors affecting disease severity will lead to the development of new tools to better control transmission and development of CL pathology in patients. Now that it is better understood that host and vector microbiota often strongly impact disease transmission and progression, it should not be surprising that a virus endemic to the same geographic regions as *L*. *major* can exert an effect on the pathology of CL [1, 9] In the future, the possibility of phleboviral co-infections should be considered in severe cases of CL, and disease treated accordingly in order to improve patient outcomes and time to disease clearance.

## Supporting information

S1 Fig Standard curve for virus titration by qRT-PCR. The standard curve for virus titration was obtained through a serial dilution of known amount of copies of the pGEM-T vector, expressing plasmid DNA for the NS-S gene, for the non-structural protein.
(PDF)

S2 Fig Transmission electron micrographs of virus particles. **A**) Transmission electron micrograph of sand fly midgut cross section. The finger-like projections, microvilli, identify the location of image as the midgut. On the right, detail of virus-like particles within intracellular compartments. **B**) TEM of virions from two different virus preparations.
(PDF)

S3 Fig SFSV induces IRF3 pathway and MAP kinase signaling. To elucidate the signaling pathways triggered by SFSV presence, B10R macrophages were infected with SFSV for 0.5 h, 1 h, and 3 h, and nuclear extracts were taken. By western blot, **A)** SFSV infection alone induced a transient increase in NF-ĸB nuclear translocation, **B)** and MAP kinase p38 and ERK1/2 phosphorylation. **C)** Additionally, phosphorylation of the IRF3 pathway was upregulated, as shown by increased phosphor-TBK and phosphor-IRF3.
(PDF)

S4 Fig *L*. *major* (co-)infection has a modulatory effect on the number and size distribution of EVs released in the peritoneal cavity 6 hours post-infection. **A)** EVs were isolated by ultracentrifugation of peritoneal cavity lavages obtained from wild-type C57BL/6 mice infected with PBS, SFSV, *L*. *major*, or co-infection with *L*. *major* and SFSV. EVs were negatively stained with uranyl acetate to reveal the ultrastructure. Their characteristic size (40-120nm) and double membrane were visible. EVs were isolated with a diameter between 60-900nm. Peak concentrations consisted of vesicles with a 100-200nm size. **B)** Total EVs isolated from the peritoneal cavity lavages were furthermore calculated. Parasitic infection, with or without SFSV, caused EVs with a wider range in diameter to be released compared to PBS or SFSV-inoculated mice.
(PDF)

S1 Table Peptide identification in *Leishmania*-infected or -uninfected samples. The detection of the peptide in either *Leishmania*-infected or–uninfected samples is indicated by an X in the respective column. The majority of peptides were identified in both *Leishmania*-infected and uninfected sandflies, indicating that the presence of virus was not dependent on the presence of the parasite. All identified peptides corresponded to those coded on the S, M, or L segments of the tripartite Phlebovirus genome.
(PDF)

## References

[1] Alvar J, Velez ID, Bern C, Herrero M, Desjeux P, Cano J, et al. Leishmaniasis worldwide and global estimates of its incidence. PLoS One. 2012;7(5):e35671. doi: 10.1371/journal.pone.0035671 22693548 PMC3365071

[2] WHO. Leishmaniasis—Fact sheet 2021 [Available from: https://www.who.int/en/news-room/fact-sheets/detail/leishmaniasis.

[3] Reithinger R, Dujardin J-C, Louzir H, Pirmez C, Alexander B, Brooker S. Cutaneous leishmaniasis. Lancet Inf Dis. 2007;7(9):581–96. 10.1016/S1473-3099(07)70209-8 17714672

[4] Ready PD. Biology of phlebotomine sand flies as vectors of disease agents. Annu Rev Entomol. 2013;58:227–50. doi: 10.1146/annurev-ento-120811-153557 23317043

[5] Rogers ME, Bates PA. Leishmania manipulation of sand fly feeding behavior results in enhanced transmission. PLoS Pathog. 2007;3(6):e91. doi: 10.1371/journal.ppat.0030091 17604451 PMC1904410

[6] Kamhawi S. Phlebotomine sand flies and Leishmania parasites: friends or foes? Trends Parasitol. 2006;22(9):439–45. doi: 10.1016/j.pt.2006.06.012 16843727

[7] Atayde VD, Aslan H, Townsend S, Hassani K, Kamhawi S, Olivier M. Exosome Secretion by the Parasitic Protozoan Leishmania within the Sand Fly Midgut. Cell Rep. 2015;13(5):957–67. doi: 10.1016/j.celrep.2015.09.058 26565909 PMC4644496

[8] Moriconi M, Rugna G, Calzolari M, Bellini R, Albieri A, Angelini P, et al. Phlebotomine sand fly-borne pathogens in the Mediterranean Basin: Human leishmaniasis and phlebovirus infections. PLoS Negl Trop Dis. 2017;11(8):e0005660. doi: 10.1371/journal.pntd.0005660 28796786 PMC5552025

[9] Alkan C, Bichaud L, de Lamballerie X, Alten B, Gould EA, Charrel RN. Sandfly-borne phleboviruses of Eurasia and Africa: epidemiology, genetic diversity, geographic range, control measures. Antiviral Res. 2013;100(1):54–74. doi: 10.1016/j.antiviral.2013.07.005 23872312

[10] (ICTV) ICoToV. “International Committee on Taxonomy of Viruses (ICTV): https://ictv.global/taxonomy/ ” 2021 [Available from: https://talk.ictvonline.org/taxonomy/p/taxonomy-history?taxnode_id=202000157.

[11] Freiberg AN, Sherman MB, Morais MC, Holbrook MR, Watowich SJ. Three-dimensional organization of Rift Valley fever virus revealed by cryoelectron tomography. J Virol. 2008;82(21):10341–8. doi: 10.1128/JVI.01191-08 18715915 PMC2573222

[12] Ikegami T, Hill TE, Smith JK, Zhang L, Juelich TL, Gong B, et al. Rift Valley Fever Virus MP-12 Vaccine Is Fully Attenuated by a Combination of Partial Attenuations in the S, M, and L Segments. J Virol. 2015;89(14):7262–76. doi: 10.1128/JVI.00135-15 25948740 PMC4473576

[13] Brett-Major DM, Claborn DM. Sand fly fever: what have we learned in one hundred years? Mil Med. 2009;174(4):426–31. doi: 10.7205/milmed-d-01-7508 19485115

[14] Ives A, Ronet C, Prevel F, Ruzzante G, Fuertes-Marraco S, Schutz F, et al. Leishmania RNA Virus Controls the Severity of Mucocutaneous Leishmaniasis. Science. 2011;331(6018):775–8. doi: 10.1126/science.1199326 21311023 PMC3253482

[15] Contreras I, Gómez MA, Nguyen O, Shio MT, McMaster RW, Olivier M. *Leishmania*-Induced Inactivation of the Macrophage Transcription Factor AP-1 Is Mediated by the Parasite Metalloprotease GP63. PLoS Pathog. 2010;6(10):e1001148. doi: 10.1371/journal.ppat.1001148 20976196 PMC2954837

[16] Atayde VD, Hassani K, da Silva Lira Filho A, Borges AR, Adhikari A, Martel C, et al. Leishmania exosomes and other virulence factors: Impact on innate immune response and macrophage functions. Cell Immunol. 2016;309:7–18. doi: 10.1016/j.cellimm.2016.07.013 27499212

[17] Filipe V, Hawe A, Jiskoot W. Critical evaluation of Nanoparticle Tracking Analysis (NTA) by NanoSight for the measurement of nanoparticles and protein aggregates. Pharm Res. 2010;27(5):796–810. doi: 10.1007/s11095-010-0073-2 20204471 PMC2852530

[18] Ready PD. Leishmaniasis emergence in Europe. Euro Surveill. 2010;15(10):19505. 20403308

[19] Hiscott J, Nguyen TLA, Arguello M, Nakhaei P, Paz S. Manipulation of the nuclear factor-kappa B pathway and the innate immune response by viruses. Oncogene. 2006;25(51):6844–67. doi: 10.1038/sj.onc.1209941 17072332 PMC7100320

[20] Isnard A, Shio MT, Olivier M. Impact of Leishmania metalloprotease GP63 on macrophage signaling. Front Cell Infect Microbiol. 2012;2:72. doi: 10.3389/fcimb.2012.00072 22919663 PMC3417651

[21] Olivier M, Gregory DJ, Forget G. Subversion mechanisms by which Leishmania parasites can escape the host immune response: a signaling point of view. Clin Microbiol Rev. 2005;18(2):293–305. doi: 10.1128/CMR.18.2.293-305.2005 15831826 PMC1082797

[22] Schamber-Reis BL, Petritus PM, Caetano BC, Martinez ER, Okuda K, Golenbock D, et al. UNC93B1 and nucleic acid-sensing Toll-like receptors mediate host resistance to infection with Leishmania major. J Biol Chem. 2013;288(10):7127–36. doi: 10.1074/jbc.M112.407684 23325805 PMC3591622

[23] Fields BN, Knipe DM, Howley PM. Structure and Organisation of Viral Genomes. 2013 2013. In: Fields Virology [Internet]. Philadelphia: Wolters Kluwer Health/Lippincott Williams & Wilkins. 6.

[24] Pothlichet J, Meunier I, Davis BK, Ting JP, Skamene E, von Messling V, et al. Type I IFN triggers RIG-I/TLR3/NLRP3-dependent inflammasome activation in influenza A virus infected cells. PLoS Pathog. 2013;9(4):e1003256. doi: 10.1371/journal.ppat.1003256 23592984 PMC3623797

[25] Chiang JJ, Davis ME, Gack MU. Regulation of RIG-I-like receptor signaling by host and viral proteins. Cytokine Growth Factor Rev. 2014;25(5):491–505. doi: 10.1016/j.cytogfr.2014.06.005 25023063 PMC7108356

[26] Nakhaei P, Genin P, Civas A, Hiscott J. RIG-I-like receptors: sensing and responding to RNA virus infection. Semin Immunol. 2009;21(4):215–22. doi: 10.1016/j.smim.2009.05.001 19539500

[27] Matte C, Olivier M. Leishmania-induced cellular recruitment during the early inflammatory response: modulation of proinflammatory mediators. J Infect Dis. 2002;185(5):673–81. doi: 10.1086/339260 11865425

[28] Charmoy M, Auderset F, Allenbach C, Tacchini-Cottier F. The prominent role of neutrophils during the initial phase of infection by Leishmania parasites. J Biomed Biotechnol. 2010;2010:719361. doi: 10.1155/2010/719361 19884987 PMC2768872

[29] Laskay T, van Zandbergen G, Solbach W. Neutrophil granulocytes—Trojan horses for Leishmania major and other intracellular microbes? Trends Microbiol. 2003;11(5):210–4. doi: 10.1016/s0966-842x(03)00075-1 12781523

[30] Cecilio P, Perez-Cabezas B, Santarem N, Maciel J, Rodrigues V, Cordeiro da Silva A. Deception and manipulation: the arms of leishmania, a successful parasite. Front Immunol. 2014;5:480. doi: 10.3389/fimmu.2014.00480 25368612 PMC4202772

[31] Zhang Y, Wu BX, Metelli A, Thaxton JE, Hong F, Rachidi S, et al. GP96 is a GARP chaperone and controls regulatory T cell functions. J Clin Invest. 2015;125(2):859–69. doi: 10.1172/JCI79014 25607841 PMC4319419

[32] Dembic Z. The cytokines of the Immune System: Elsevier; 2015.

[33] Goswami R, Kaplan MH. A brief history of IL-9. J Immunol. 2011;186(6):3283–8. doi: 10.4049/jimmunol.1003049 21368237 PMC3074408

[34] Hirano T, Yasukawa K, Harada H, Taga T, Watanabe Y, Matsuda T, et al. Complementary DNA for a novel human interleukin (BSF-2) that induces B lymphocytes to produce immunoglobulin. Nature. 1986;324(6092):73–6. doi: 10.1038/324073a0 3491322

[35] Yoshimura T. The chemokine MCP-1 (CCL2) in the host interaction with cancer: a foe or ally? Cell Mol Immunol. 2018;15(4):335–45. doi: 10.1038/cmi.2017.135 29375123 PMC6052833

[36] Sarafi MN, Garcia-Zepeda EA, MacLean JA, Charo IF, Luster AD. Murine monocyte chemoattractant protein (MCP)-5: a novel CC chemokine that is a structural and functional homologue of human MCP-1. J Exp Med. 1997;185(1):99–109. doi: 10.1084/jem.185.1.99 8996246 PMC2196097

[37] Belkaid Y, Piccirillo CA, Mendez S, Shevach EM, Sacks DL. CD4+CD25+ regulatory T cells control Leishmania major persistence and immunity. Nature. 2002;420(6915):502–7. doi: 10.1038/nature01152 12466842

[38] Liu M, Guo S, Hibbert JM, Jain V, Singh N, Wilson NO, et al. CXCL10/IP-10 in infectious diseases pathogenesis and potential therapeutic implications. Cytokine Growth Factor Rev. 2011;22(3):121–30. doi: 10.1016/j.cytogfr.2011.06.001 21802343 PMC3203691

[39] Guo XK, Zhao WQ, Kondo C, Shimojo N, Yamashita K, Aoki T, et al. Tissue inhibitors of metalloproteinases-1 (TIMP-1) and -2(TIMP-2) are major serum factors that stimulate the TIMP-1 gene in human gingival fibroblasts. Biochim Biophys Acta. 2006;1763(3):296–304. doi: 10.1016/j.bbamcr.2006.02.012 16631927

[40] Kobuch J, Cui H, Grunwald B, Saftig P, Knolle PA, Kruger A. TIMP-1 signaling via CD63 triggers granulopoiesis and neutrophilia in mice. Haematologica. 2015;100(8):1005–13. doi: 10.3324/haematol.2014.121590 26001794 PMC5004415

[41] Takei T, Ando H., Tsutsui K. Granulocyte Colony-Stimulating Factor. Handbook of Hormones: Elsevier; 2016.

[42] Rath CT, Schnellrath LC, Damaso CR, de Arruda LB, Vasconcelos P, Gomes C, et al. Amazonian Phlebovirus (Bunyaviridae) potentiates the infection of Leishmania (Leishmania) amazonensis: Role of the PKR/IFN1/IL-10 axis. PLoS Negl Trop Dis. 2019;13(6):e0007500. doi: 10.1371/journal.pntd.0007500 31216268 PMC6602282

[43] Wuerth JD, Habjan M, Wulle J, Superti-Furga G, Pichlmair A, Weber F. NSs Protein of Sandfly Fever Sicilian Phlebovirus Counteracts Interferon (IFN) Induction by Masking the DNA-Binding Domain of IFN Regulatory Factor 3. J Virol. 2018;92(23). doi: 10.1128/JVI.01202-18 30232186 PMC6232482

[44] Rossi M, Castiglioni P, Hartley MA, Eren RO, Prevel F, Desponds C, et al. Type I interferons induced by endogenous or exogenous viral infections promote metastasis and relapse of leishmaniasis. Proc Natl Acad Sci U S A. 2017;114(19):4987–92. doi: 10.1073/pnas.1621447114 28439019 PMC5441690

[45] Gowen BB, Hoopes JD, Wong MH, Jung KH, Isakson KC, Alexopoulou L, et al. TLR3 deletion limits mortality and disease severity due to Phlebovirus infection. J Immunol. 2006;177(9):6301–7. doi: 10.4049/jimmunol.177.9.6301 17056560

[46] Belkaid Y, Mendez S, Lira R, Kadambi N, Milon G, Sacks D. A natural model of Leishmania major infection reveals a prolonged "silent" phase of parasite amplification in the skin before the onset of lesion formation and immunity. J Immunol. 2000;165(2):969–77. doi: 10.4049/jimmunol.165.2.969 10878373

[47] Kumar R, Bumb RA, Salotra P. Evaluation of localized and systemic immune responses in cutaneous leishmaniasis caused by Leishmania tropica: interleukin-8, monocyte chemotactic protein-1 and nitric oxide are major regulatory factors. Immunol. 2010;130(2):193–201. doi: 10.1111/j.1365-2567.2009.03223.x 20102417 PMC2878464

[48] Nylen S, Eidsmo L. Tissue damage and immunity in cutaneous leishmaniasis. Par Immunol. 2012;34(12):551–61. doi: 10.1111/pim.12007 23009296

[49] Carty M, Bowie AG. Recent insights into the role of Toll-like receptors in viral infection. Clin Exp Immunol. 2010;161(3):397–406. doi: 10.1111/j.1365-2249.2010.04196.x 20560984 PMC2962956

[50] Takeuchi O, Akira S. Innate immunity to virus infection. Immunol Rev. 2009;227(1):75–86. doi: 10.1111/j.1600-065X.2008.00737.x 19120477 PMC5489343

[51] Yamada S, Shimojima M, Narita R, Tsukamoto Y, Kato H, Saijo M, et al. RIG-I-Like Receptor and Toll-Like Receptor Signaling Pathways Cause Aberrant Production of Inflammatory Cytokines/Chemokines in a Severe Fever with Thrombocytopenia Syndrome Virus Infection Mouse Model. J Virol. 2018;92(13). 10.1128/JVI.02246-17 PMC6002725 29643242

[52] Roberts KK, Hill TE, Davis MN, Holbrook MR, Freiberg AN. Cytokine response in mouse bone marrow derived macrophages after infection with pathogenic and non-pathogenic Rift Valley fever virus. J Gen Virol. 2015;96(Pt 7):1651–63. doi: 10.1099/vir.0.000119 25759029 PMC4635452

