# Supplementary material for: Sandfly Fever Sicilian Virus-Leishmania major co-infection modulates innate inflammatory response favoring myeloid cell infections and skin hyperinflammation
Source: PLoS Negl Trop Dis. 2021 Jul 26;15(7):e0009638. doi: 10.1371/journal.pntd.0009638 (PMC8341699; doi:10.1371/journal.pntd.0009638)
Supplement: S1 Fig — The standard curve for virus titration was obtained through a serial dilution of known amount of copies of the pGEM-T vector, expressing plasmid DNA for the NS-S gene, for the non-structural protein. (PDF) [file pntd.0009638.s001.pdf]

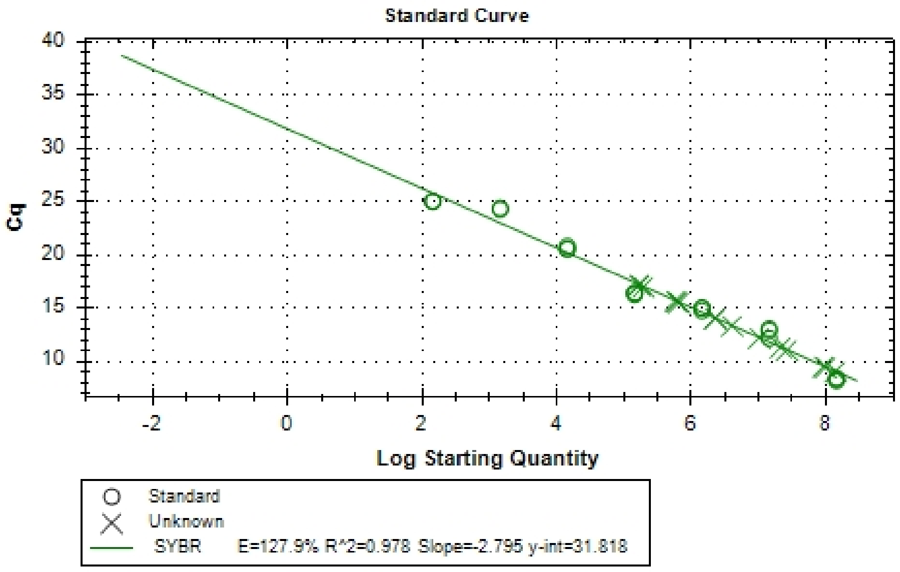

S1 Fig: Standard curve for virus titration by qRT-PCR. The standard curve for virus titration was obtained through a serial dilution of known amount of copies of the pGEM-T vector, expressing plasmid DNA for the NS-S gene, for the non-structural protein.
