# Supplementary material for: Sandfly Fever Sicilian Virus-Leishmania major co-infection modulates innate inflammatory response favoring myeloid cell infections and skin hyperinflammation
Source: PLoS Negl Trop Dis. 2021 Jul 26;15(7):e0009638. doi: 10.1371/journal.pntd.0009638 (PMC8341699; doi:10.1371/journal.pntd.0009638)
Supplement: S2 Fig — A) Transmission electron micrograph of sand fly midgut cross section. The finger-like projections, microvilli, identify the location of image as the midgut. On the right, detail of virus-like particles within intracellular compartments. B) TEM of virions from two different virus preparations. (PDF) [file pntd.0009638.s002.pdf]

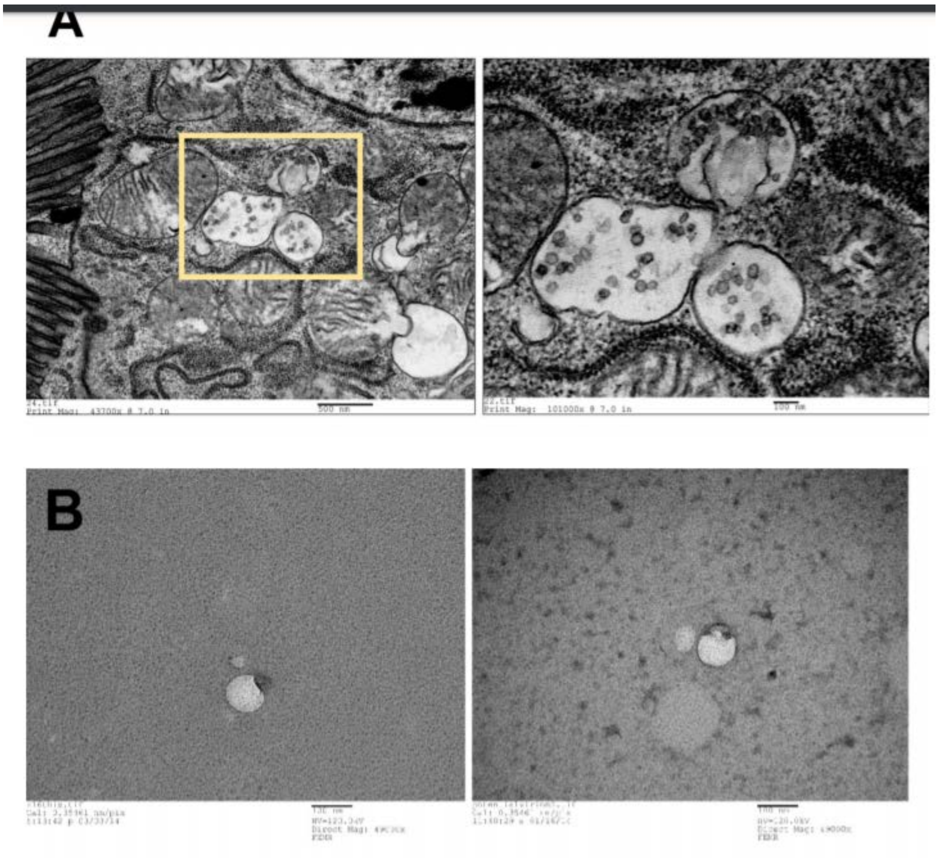

S2 Fig: Transmission electron micrographs of virus particles. A) Transmission electron micrograph of sand fly midgut cross section. The finger-like projections, microvilli, identify the location of image as the midgut. On the right, detail of virus-like particles within intracellular compartments. **B**) TEM of virions from two different virus preparations.
