# Supplementary material for: Sandfly Fever Sicilian Virus-Leishmania major co-infection modulates innate inflammatory response favoring myeloid cell infections and skin hyperinflammation
Source: PLoS Negl Trop Dis. 2021 Jul 26;15(7):e0009638. doi: 10.1371/journal.pntd.0009638 (PMC8341699; doi:10.1371/journal.pntd.0009638)
Supplement: S3 Fig — To elucidate the signaling pathways triggered by SFSV presence, B10R macrophages were infected with SFSV for 0.5 h, 1 h, and 3 h, and nuclear extracts were taken. By western blot, A) SFSV infection alone induced a transient increase in NF-ĸB nuclear translocation, B) and MAP kinase p38 and ERK1/2 phosphorylation. C) Additionally, phosphorylation of the IRF3 pathway was upregulated, as shown by increased phosphor-TBK and phosphor-IRF3. (PDF) [file pntd.0009638.s003.pdf]

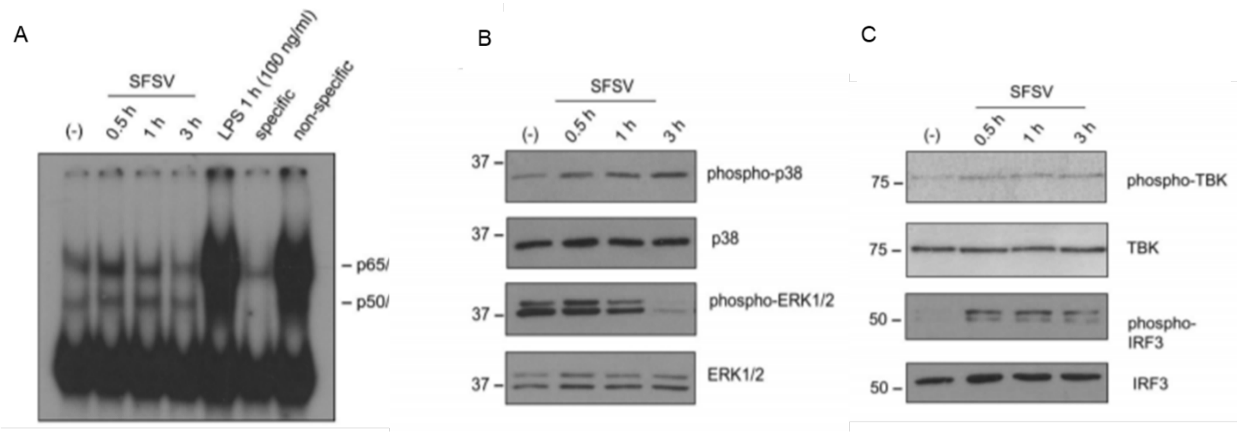

S3 Fig: SFSV induces IRF3 and MAP kinase signaling pathways. To elucidate the signaling pathways triggered by SFSV presence, B10R macrophages were infected with SFSV for 0.5 h, 1 h, and 3 h, and total cell protein and nuclear extracts were taken. Band-Shift Assay, **A)** SFSV infection alone induced a transient increase in NF-kB nuclear translocation. By western blot were monitored, **B)** MAP kinase p38 and ERK1/2 phosphorylation, and, **C)** TBK and IRF3 phosphorylation.
