# Supplementary material for: Sandfly Fever Sicilian Virus-Leishmania major co-infection modulates innate inflammatory response favoring myeloid cell infections and skin hyperinflammation
Source: PLoS Negl Trop Dis. 2021 Jul 26;15(7):e0009638. doi: 10.1371/journal.pntd.0009638 (PMC8341699; doi:10.1371/journal.pntd.0009638)
Supplement: S4 Fig — A) EVs were isolated by ultracentrifugation of peritoneal cavity lavages obtained from wild-type C57BL/6 mice infected with PBS, SFSV, L. major, or co-infection with L. major and SFSV. EVs were negatively stained with uranyl acetate to reveal the ultrastructure. Their characteristic size (40-120nm) and double membrane were visible. EVs were isolated with a diameter between 60-900nm. Peak concentrations consisted of vesicles with a 100-200nm size. B) Total EVs isolated from the peritoneal cavity lavages were furthermore calculated. Parasitic infection, with or without SFSV, caused EVs with a wider range in diameter to be released compared to PBS or SFSV-inoculated mice. (PDF) [file pntd.0009638.s004.pdf]

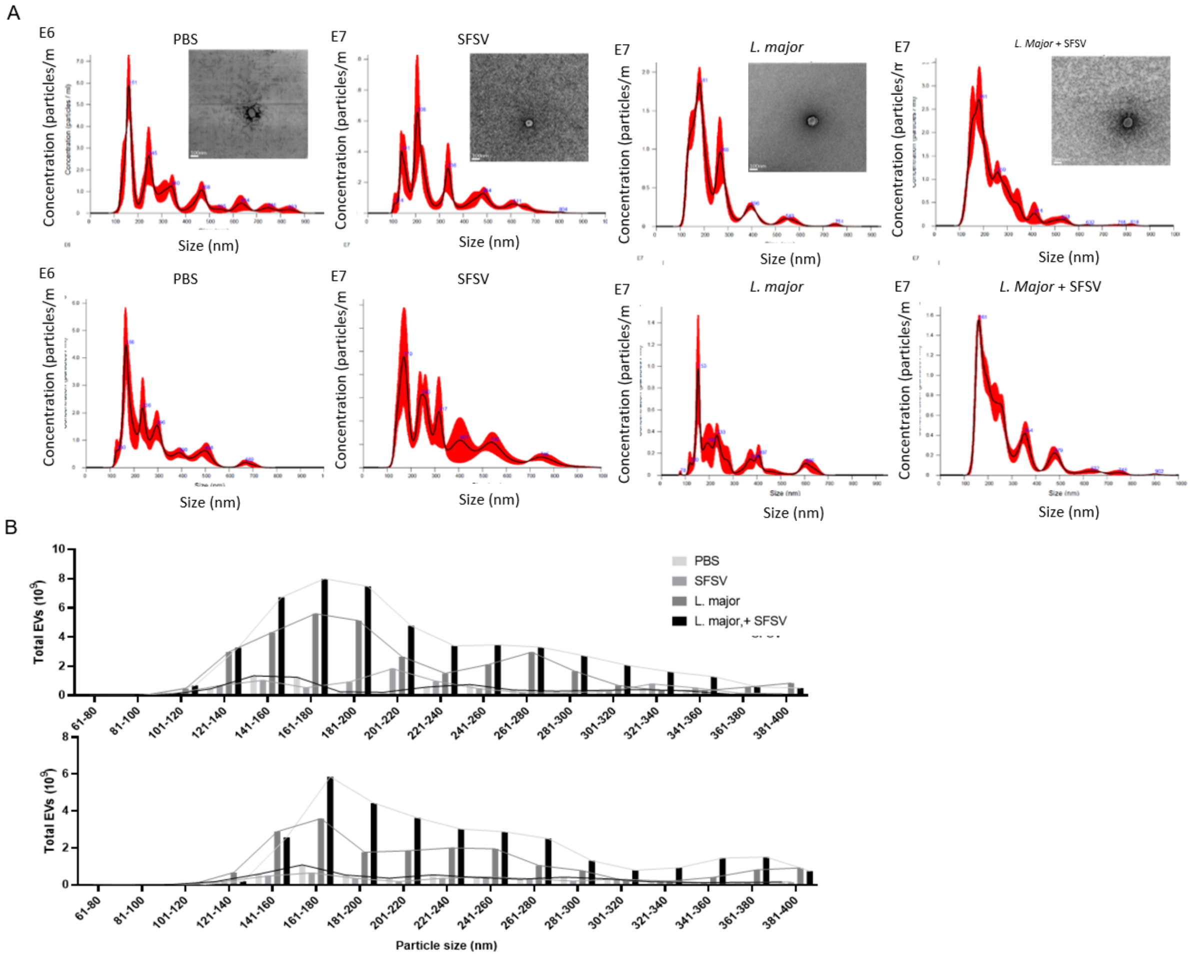

S4 Fig: *L. major* (co-)infection has a modulatory effect on the number and size distribution of EVs released in the peritoneal cavity 6 hours post-infection. A) EVs were isolated by ultracentrifugation of peritoneal cavity lavages obtained from wild-type C57BL/6 mice infected with PBS, SFSV, *L. major*, or co-infection with *L. major* and SFSV. EVs were negatively stained with uranyl acetate to reveal the ultrastructure. Their characteristic size (40-120nm) and double membrane were visible. EVs were isolated with a diameter between 60-900nm. Peak concentrations consisted of vesicles with a 100-200nm size. **B)** Total EVs isolated from the peritoneal cavity lavages were furthermore calculated. Parasitic infection, with or without SFSV, caused EVs with a wider range in diameter to be released compared to PBS or SFSV-inoculated mice.
