# Supplementary material for: Sandfly Fever Sicilian Virus-Leishmania major co-infection modulates innate inflammatory response favoring myeloid cell infections and skin hyperinflammation
Source: PLoS Negl Trop Dis. 2021 Jul 26;15(7):e0009638. doi: 10.1371/journal.pntd.0009638 (PMC8341699; doi:10.1371/journal.pntd.0009638)
Supplement: S1 Table — The detection of the peptide in either Leishmania-infected or–uninfected samples is indicated by an X in the respective column. The majority of peptides were identified in both Leishmania-infected and uninfected sandflies, indicating that the presence of virus was not dependent on the presence of the parasite. All identified peptides corresponded to those coded on the S, M, or L segments of the tripartite Phlebovirus genome. (PDF) [file pntd.0009638.s005.pdf]

Identified proteins	Putative species	Accession no.	UNINFECTED	INFECTED
Nucleocapsid	[Phlebovirus sp. Co Ar 171616]	gi|146336901	X	X
Nucleocapsid	[Precarious point virus]	gi|342360103		X
Glycoprotein polyprotein	[Heartland virus]	gi|399207768		X
Glycoprotein	[Armero virus]	gi|328933902	X	X
Polyprotein	[Mucura virus]	gi|327178166	X	
Polyprotein	[Toscana virus]	gi|343454840	X	X
Polyprotein	[Sand fly fever Turkey virus]	gi|329738745	X	X
Polyprotein	[Itaituba virus]	gi|327178164	X	X
Polyprotein	[Nique virus]	gi|327178170	X	X
Polymerase	[Phlebovirus GGP-2011a]	gi|342360114	X	X
Polymerase	[Rift Valley fever virus]	gi|433287020-R		X
RNA polymerase	[SFTSV]	gi|387861575	X	X
RNA dependent RNA polymerase	[Uukuniemi virus]	gi|333494494		X
RNA-dependant RNA polymerase	[Huaiyangshan virus]	gi|327177385-R	X	X
Polymerase	[Toscana virus]	gi|167594025	X	X
Polymerase	[Sand fly fever Naples-like virus]	gi|342360093	X	X
Polymerase	[Sand fly fever Turkey virus]	gi|329738747	X	X
L protein	[Echarate virus]	gi|327178186	X	X
L protein	[Granada virus]	gi|308197165	X	X

S1 Table: Peptide identification in *Leishmania*-infected or -uninfected samples. The detection of the peptide in either *Leishmania*-infected or –uninfected samples is indicated by an X in the respective column. The majority of peptides were identified in both *Leishmania*-infected and uninfected sandflies, indicating that the presence of virus was not dependent on the presence of the parasite. All identified peptides corresponded to those coded on the S, M, or L segments of the tripartite Phlebovirus genome.
